# Differential regulation of oxidative stress, microbiota-derived, and energy metabolites in the mouse brain during sleep

**DOI:** 10.1177/0271678X211033358

**Published:** 2021-07-22

**Authors:** Theodosia Vallianatou, Weifeng Lin, Nicholas B Bèchet, Mario SP Correia, Nagesh C Shanbhag, Iben Lundgaard, Daniel Globisch

**Affiliations:** 1 Department of Chemistry-BMC, Science for Life Laboratory, Uppsala University, Uppsala, Sweden; 2 Department of Experimental Medical Science, 5193 Lund University, Lund University, Lund, Sweden; 3 Wallenberg Centre for Molecular Medicine, 5193 Lund University, Lund University, Lund, Sweden

**Keywords:** Brain metabolism, energy metabolism, gut-brain axis, metabolomics, oxidative stress

## Abstract

Sleep has evolved as a universal core function to allow for restorative biological processes. Detailed knowledge of metabolic changes necessary for the sleep state in the brain is missing. Herein, we have performed an in-depth metabolic analysis of four mouse brain regions and uncovered region-specific circadian variations. Metabolites linked to oxidative stress were altered during sleep including acylcarnitines, hydroxylated fatty acids, phenolic compounds, and thiol-containing metabolites. These findings provide molecular evidence of a significant metabolic shift of the brain energy metabolism. Specific alterations were observed for brain metabolites that have previously not been associated with a circadian function including the microbiome-derived metabolite ergothioneine that suggests a regulatory function. The pseudopeptide *β*-citryl-glutamate has been linked to brain development and we have now discovered a previously unknown regioisomer. These metabolites altered by the circadian rhythm represent the foundation for hypothesis-driven studies of the underlying metabolic processes and their function.

## Introduction

The impact of sleep on brain homeostasis and healthy neuronal activity is an increasingly investigated field in neuroscience. While sleep is a biological state that can increase the vulnerability to external threats such as limited defense against predation, it is highly conserved among species. Despite this negative evolutionary development, sleep has an essential role in brain physiology.^1^ While this role is not yet fully understood, sleep has been associated with restorative functions in the brain and memory consolidation. Several sleep deprivation studies have further demonstrated the detrimental effects of reduced sleep on cognitive performance.^2^ Furthermore, the recent glymphatic hypothesis has shown the importance of sleep for the clearance process of potentially neurotoxic compounds from the central nervous system (CNS).^3–5^ It is hypothesized that some molecules accumulate during the awake phase. In addition, it has been demonstrated that instead of an energetical “shut down”, the brain performs a shift in metabolic processes during sleep.^6^ Specifically, the relationship between glucose consumption and oxidative metabolism in sleep is significantly different compared to wakefulness.^6,7^ While many studies have focused on the profoundly altered neuronal activity during sleep, comprehensive metabolic profiles of the sleeping and conscious brain are limited. These studies are crucial to provide insights into the metabolic function and underlying mechanisms during sleep such as changes in energy consumption or oxidative processes.

Metabolomics is a powerful tool to reveal molecular processes that are prominent during the sleep state. The brain is constituted by highly inhomogeneous regions that are anatomically and functionally distinct. Comparative analysis of multiple brain regions can provide a comprehensive understanding of complex brain functions and only a limited number of studies has been reported.^8,9^ Our method of choice for investigation of metabolites was mass spectrometry-based untargeted metabolomics that has been extensively applied in elucidating metabolic processes in different (patho)physiological states.^10^ In sleep research, metabolomics studies have highlighted small molecules such as tricarboxylic acid (TCA) cycle intermediates, methionine metabolism, other amino acids, and fatty acids to be significantly altered depending on the arousal state.^11^ These studies mainly focused on circulating plasma metabolites as well as sleep deprivation and thus not on normal sleep.^11,12^ However, systemic concentration changes in plasma are not representative of the CNS status as the brain entry and clearance of metabolites is regulated by a highly restrictive formation of the brain capillary endothelia, the blood-brain barrier (BBB).^13,14^

In the present study, global metabolomics using ultra performance liquid chromatography coupled with tandem mass spectrometry (UPLC-MS/MS) was applied to uncover sleep-induced metabolic changes in the four major mouse brain regions: cortex, hippocampus, midbrain, and cerebellum. Our findings demonstrate the regional effect on multiple metabolite classes including metabolites altered during sleep such as acylcarnitines, amino acids and their modifications, nucleotides among other metabolic intermediates. These findings were further corroborated by global metabolomics analysis of plasma and liver tissue samples from the same animals to identify systemic changes. The comprehensive results from this study build the basis for future mechanistic studies during sleep, metabolite clearance and mapping metabolic processes in the brain.

## Materials and methods

### Chemicals

Solvents and reagents were purchased from Sigma-Aldrich or Fisher Scientific and were used without further purification. All synthesized compounds were ≥95% pure as determined by NMR. NMR spectra were recorded on an Agilent 400 MHz spectrometer (^1^H-NMR: 399.97 MHz and ^13^C NMR: 100.58 MHz). Chemical shifts are reported in parts per million (ppm) on the δ scale from an internal standard. Multiplicities are abbreviated as follows: s = singlet, d = doublet, t = triplet, q = quartet, and m = multiplet. Authentic standards were also purchased from Sigma-Aldrich or Fisher Scientific. The in-house built metabolite library was obtained from MetaSci. Mass spectrometry grade solvents were used for UPLC-ESI-MS analysis.

### Animal experiments

Adult male C57BL/6 mice (Janvier Labs) were housed in standard laboratory conditions with a 12-hour dark light cycle with ad libitum access to water and food. All experiments were performed according to ethical approval from the Malmö-Lund Ethical Committee on Animal Research (Dnr 5.8.18- 08269/2019) and conducted according to the CODEX guidelines by the Swedish Research Council, Directive 2010/63/EU of the European Parliament on the protection of animals used for scientific purposes and Regulation (EU) 2019/1010 on the alignment of reporting obligations. This study complies with the ARRIVE guidelines.^15^ Animals were sacrificed by cervical dislocation at either 10AM or 10PM and subsequently decapitated, at an age of 8 weeks. Trunk blood was collected and plasma samples were prepared by centrifugation. Brains were rapidly extracted (<2 minutes) after dislocation and dissected to isolate cortex, hippocampus, midbrain and cerebellum. Brain regions were then placed in Eppendorf tubes and snap frozen at −80°C in liquid nitrogen. Liver tissue was extracted from the same animals and snap frozen at −80°C in liquid nitrogen.

### Tissue processing and sample preparation

The brain regions were weighed and transferred into beads-containing vials. Methanol:water (80:20) was added approximately at a concentration of 4 µL/mg brain tissue to every sample. As internal standard (I.S.), a mixture of C-13 isotopically labeled tyrosine (5 µg/mL), phenylalanine (10 µg/mL) and valine (30 µg/mL). The volume of the I.S. mixture added to every sample was adjusted according to the corresponding sample weight, with a minimum of 10 µL for the lowest weight sample. The homogenization was performed in a Lysing matrix D instrumentation (MP Biomedicals) in dry ice at a cycle of 20 s shaking (4 m/s) and 30 s performed three times. Samples were collected, precipitated on ice for 1 h and centrifuged at 13,400 rpm for 5 min. The supernatant was collected and dried under vacuum on a Speedvac and subsequently stored in -20°C for a maximum of three days prior to analysis. Samples were re-suspended with water:acetonitrile (95:5) prior to UPLC-MS/MS analysis, at a volume normalized to the sample weight. Quality control (QC) samples were prepared by 5 µL aliquots from all samples. The same process was followed for the preparation of the liver samples that were analyzed via UPLC-MS in a separate sequence.

For the preparation of plasma samples, the same I.S. mixture was added to each plasma aliquot (50 µL). QC samples were prepared by 5 µL aliquots from all samples after thawing on ice. Sample preparation was performed on ice. LC-MS grade methanol (1:4 ratio of sample:methanol) was added and the mixture was vortexed before being cooled to -20°C for 1 hour. The samples were then centrifuged (5 min, 18,620 g, 4°C) and the supernatant was isolated and lyophilized on a Speedvac. The samples were stored in -20°C until the UPLC-MS analysis. The samples were reconstituted in 50 µL water: acetonitrile (95:5) and centrifuged again (5 min, 18,620 g, 4°C). The supernatant was transferred to LC-MS vials.

### UPLC mass spectrometry

The UPLC-MS/MS analysis was performed in a SYNAPT G2-S High-Definition Mass Spectrometer (HDMS) using an electrospray ionization (ESI) source with an AQCUITY UPLC I-class system and equipped with a Waters ACQUITY UPLC® HSS T3 column (1.8 µm, 100 × 2.1 mm). Water with 0.1% formic acid was used as mobile phase A and methanol with 0.1% formic acid was used as mobile phase B. The column temperature was kept at 40°C, and the autosampler at 6°C. The flow rate was set to 0.2 mL/min. The gradient used was as follows: 0–8.5 min, 0–100% B; 8.5–10 min, 100% B; 10–11 min,100–0% B; 10–15 min, 0% B.

The system was controlled using the MassLynx software package v 4.1 from Waters. High-resolution mass spectra were acquired in positive and negative ionization mode, at a mass range of m/z 50-1500. Data acquisition was performed in MSE mode. The samples were injected to the UPLC-MS system in a randomized order with QC samples injected in the beginning and end of the sample list in both ionization modes, as well as after every eight samples (7 QCs in each ionization mode in total).

### Identification of metabolites

Significant features and molecules of interest were primarily annotated by databases (www.hmdb.ca, https://metlin.scripps.edu/) based on their *m/z* value and given the high mass accuracy provided by the mass analyzer. Subsequently, in-house built standard library or purchased standards, measured in the same UPLC-MS/MS system, were used for the assignment of the retention time (rt). Finally, tandem MS experiments were performed in brain tissue samples in positive or negative ionization mode with CID of 10–30 eV, depending on the analyte, and the product ion spectra were compared to the corresponding standards.

### Data analysis

The chromatograms and mass spectra were processed using the XCMS R package for peak alignment and retention time correction,^16,17^ in both positive and negative ionization mode. From the corresponding feature lists obtained from the software, features with intensities > 20,000 ion count, rt > 1 min and %CV of the QCs < 30 were selected for further statistical analysis, as considerably higher than noise. The final data included 17,297 features in positive and 7,578 features in negative ionization mode measured in four different brain regions (CBL, CTX, HC and MDB) of both groups (sleep-wake, N = 6 per group). The intensities of the included internal standards and the QC samples were plotted against the UPLC-MS/MS sample injection order to evaluate the stability and performance of the experimental set over time.

An overview of the data was provided by principal component analysis (PCA), prior to which the data was autoscaled using the metabolomics platform www.metaboanalyst.ca. The normality of the test statistics and *P* values were evaluated using the same platform and the data were distributed normally. For the hypothesis testing, two-tailed *t*-test was applied in metabolites extracted from every region (CBL, CTX, HC, MDB) for detecting consciousness state (sleep-wake) differences. The same approach was followed for the plasma and liver sample analysis. Owing to the large size of imported features, the significance of the results was cross validated with two-way ANOVA (factors: brain regions, sleep/wake) with adjustment for multiple comparisons (Supplementary Table 8).

### Synthesis of *β*-citryl-glutamate (1)

*Synthesis of 1,5-dimethyl citrate (****3****)*: 1,5-Dimethyl citrate was prepared following a literature procedure.^18^ Citric acid (10.0 g, 52.1 mmol) was dissolved in methanol (100 mL) followed by slow addition of 98% sulfuric acid (1 mL). The mixture was refluxed for 1 h then allowed to cool to room temperature before cold water (50 mL) was added under stirring. The solution was neutralized with calcium carbonate. The suspension was filtered and the filtrate evaporated to dryness in vacuo. This yielded colorless crystals, which was redissolved in cold water (100 mL), filtered to remove traces of insoluble salts and the filtrate was acidified to pH 4.5 with a 5 M hydrochloric acid solution. The resulting white precipitate was filtered off to yield the desired product (3.50 g, 15.9 mmol, 31%).

*Synthesis of dimethyl (S)-3-((1,5-diethoxy-1,5-dioxopentan-2-yl)carbamoyl)-3-hydroxypentanedioate (Protected β-citrylglutamate)* (**4**): To the solution of 1,5-dimethyl citrate (**3**, 100 mg, 454.5 µmol, 1 equiv.) in dichloromethane (10 mL), diethyl glutamate (**2**, 100 mg, 492.6 µmol, 1.1 equiv.), HBTU (234 mg, 590.9 µmol, 1.3 equiv.), HOBT (83.4 mg, 590.9 µmol, 1.3 equiv.) and DIPEA (232 µL, 1.36 mmol, 3 equiv.) were sequentially added. The reaction mixture was stirred at room temperature for 16 h. Upon full consumption of the starting material by TLC monitoring, the reaction mixture was quenched with saturated aqueous NaHCO_3_ (15 mL). The reaction mixture was extracted with DCM (3 × 10 mL), and the combined organic layers were washed with aqueous NaHCO_3_ (2 × 5 mL), Na_2_CO_3_ (2 × 5 mL) and brine (2 × 10 mL). After evaporation, the residue was purified by flash chromatography on silica gel using a gradient of 2% MeOH in chloroform to afford compound 4 (128.3 mg, 316 µmol, 69%).^1^H NMR (401 MHz, CD_3_OD) δ (ppm) = 4.48 (dd, J = 8.8, 4.9 Hz, 1H), 4.21 (q, J = 7.1 Hz, 2H), 4.13 (q, J = 7.1 Hz, 2H), 3.65 (d, J = 6.2 Hz, 6H), 3.02–2.68 (m, 4H), 2.52–2.38 (m, 2H), 2.28–2.15 (m, 1H), 2.09–1.94 (m, 1H), 1.27 (dt, J = 17.7, 7.1 Hz, 6H). ^13^C NMR (101 MHz, CD_3_OD) δ (ppm) 175.80, 174.54, 172.77, 171.92, 171.78, 75.19, 62.61, 61.64, 52.99, 52.24, 52.20, 44.14, 43.77, 30.96, 27.98, 14.50, 14.45. HRMS (ESI+) m/z for C_17_H27O_10_N [M + H]^+^ calculated 406.1708, found 406.1719.

*Synthesis of β-citrylglutamate (****1****)*: To a solution of compound **4** (6.5 mg, 16.0 µmol) in dioxane (2 mL), 5 M HCl solution (2 mL) was added, and the reaction mixture was stirred at 55°C for 5 h. The reaction was neutralized with Na_2_CO_3_. After the removal of solvent, the residue was purified by flash chromatography on C18-reversed phase silica gel using 50% MeOH in H_2_O to yield *β*-citrylglutamate (**1**, 2.8 mg, 8.7 µmol, 55%). ^1^H NMR (401 MHz, CD_3_OD) δ (ppm) = 4.48 (dd, J = 8.4, 4.8 Hz, 1H), 2.98–2.66 (m, 4H), 2.45 (td, J = 8.6, 8.2, 6.5 Hz, 2H), 2.29–2.18 (m, 1H), 2.09–1.97 (m, 1H), ^13^C NMR (101 MHz, CD_3_OD) δ (ppm) 176.29, 175.15, 174.39, 173.61, 173.33, 75.15, 52.97, 43.70, 43.39, 30.69, 27.77. HRMS (ESI+) m/z for C_11_H_15_O_10_N [M + H]^+^ calculated 322.0769, found 322.0768.

## Results

### Regional brain metabolomics

**Figure 1.**
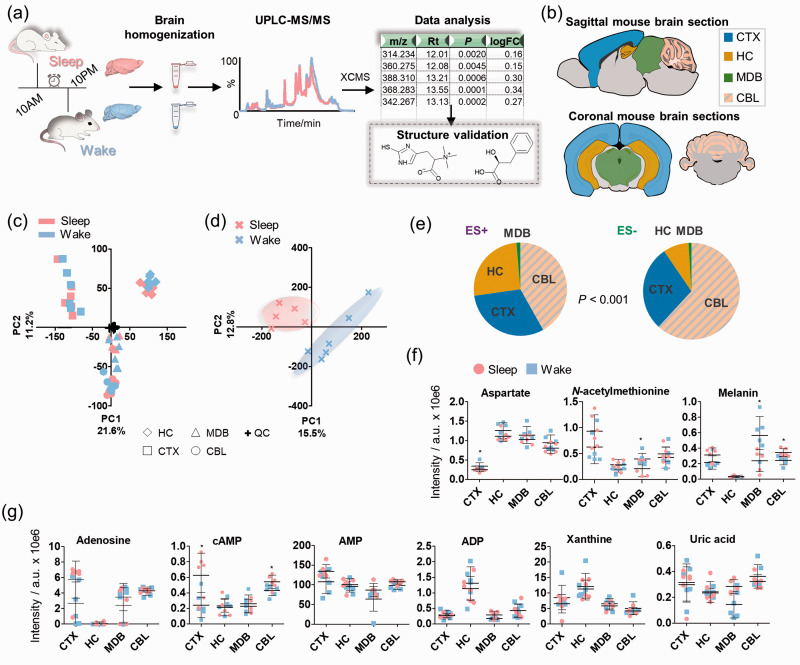
Overview of the experimental workflow and metabolomics analysis. (a) Study design; (b) The investigated brain regions in sagittal and coronal mouse brain tissue sections; (c) Principal component analysis on the different mouse brain regions (features from positive and negative ionization mode); (d) Scores plot of the principal component analysis on the animals based on the intensities of the included features per region in both positive and negative ionization mode. One sample was excluded from the sleep group due to one missing brain region sample; (e) Pie chart of the significant features (*t*-test; *P* < 0.001) per region in positive (ES+) and negative (ES−) ionization mode (N = 6); (f) Mass spectrometric intensities of selected metabolites in the investigated brain regions in sleep and wake state (N = 6); (g) Mass spectrometric intensities of purinergic metabolites in the investigated brain regions in sleep and wake state (N = 6); Error bars: standard deviation (SD); two-tailed unpaired *t*-test: *P < 0.05, **P < 0.01. CBL: cerebellum; CTX: cortex; HC: hippocampus; MDB: midbrain.

Brain region-specific metabolomics was conducted to identify regional differences in metabolism at two distinct states of the circadian rhythm. For our study, mice sacrificed 3 hours into the light and dark phase corresponding to their subjective night (referred to as sleep state) and day (referred to as awake state), respectively (N = 6 in each group; Figure 1(a)). Afterwards, the brain regions collected for the analysis were cortex (CTX), hippocampus (HC), midbrain (MDB), and cerebellum (CBL) (Figure 1(b)). Plasma and liver samples were also collected for control analysis to identify systemic metabolite changes. Each brain region was weighed and then homogenized using ceramic beads. Quality control (QC) samples were prepared after homogenization (Supplementary Figure 1). Metabolites from each biological replica of every examined brain region were extracted separately following standard procedures and spiking of an isotopically labelled internal standard.^19,20^ The samples were analyzed by UPLC-MS/MS with a randomized sample sequence in negative and positive ionization mode. The volume during reconstitution was adjusted to the weight of each brain region and the samples were analyzed by UPLC-MS/MS (Supplementary Table 1).

The UPLC-MS data for the brain region samples were processed with R using the XCMS metabolomics framework.^17^ To obtain a global overview of the data, the mass spectrometric features were considered that fit the following general criteria: retention time > 1 min and average intensity > 20,000 ion counts. Unsupervised principal component analysis (PCA) of the combined features from positive and negative ionization mode yielded separation of each distinct brain region as well as separation of the sleep and wake state for each brain region. The QC samples were centered as expected for high quality metabolomics data (Figure 1(c)). The highest variation was observed for the cortex compared with the hippocampus (PC1), while an anatomical differentiation among cerebrum (CTX and HC), midbrain (MDB) and cerebellum (CBL) was displayed (PC2). Furthermore, MDB and CBL clustered with high proximity, which could be a result from their anatomical interconnection (Supplementary Figure 2). Unsupervised analysis revealed strong separation of the sleep-wake samples based on the metabolic differences between the subjective day and night phase (Figure 1(d)). The most significantly altered features for each brain region identified that cerebellum was associated with the most sleep-specific metabolic alterations, while the opposite was observed for the midbrain (Figure 1(e)).

As a proof-of-concept analysis, we investigated selected brain molecules that have previously been linked to brain functions. The region-specific responses associated with the sleep-wake cycle are highlighted for the explored brain regions of both groups and demonstrate similarities and differences of these selected metabolites in each brain region (Supplementary Figure 3). While most of these metabolites were identified with varying levels in different brain regions, they displayed the same levels in the sleep and awake sample set. We also observed statistically significant differences in single brain regions (Figure 1(f); Table 1). Aspartate, which is an excitatory amino acid, was elevated during the wake phase in CTX, while *N*-acetylmethionine was significantly depleted in MDB during sleep. Melanin is a downstream metabolic product of the dopamine pathway and was significantly elevated in the wakeful state in CBL and reduced in MDB.^21^ Furthermore, the endogenous sleep-inducing molecule adenosine and its cyclic monophosphate derivative (cAMP) were also detected at higher levels during sleep in CTX and CBL (Figure 1(g)). Based on this finding, we investigated other analogues of adenosine including adenosine monophosphate (AMP), adenosine diphosphate (ADP) as well as guanosine and guanosine monophosphate, which did not show any variation across the sleep-wake cycle (Figure 1(g) and Supplementary Figure 3). Other down-stream products of the purinergic metabolism (e.g. xanthine and uric acid) were also unaltered. Purinergic signaling, including the release of adenosine triphosphate (ATP), is implicated in sleep and arousal regulation.^22–24^ However, rapid degradation usually impedes the detection of ATP via LC-MS approaches.^25^ Complete discrimination of the two sample sets was identified based on the top twenty metabolites (Supplementary Figures 3-9, Supplementary Tables 2-3). The reproducibility of this regional analysis of common metabolites lays the foundation to characterize unknown metabolic alterations specific for the sleep-wake cycle.

**Table 1.** Significant sleep-wake effects on brain metabolites in specific brain regions. Two tailed unpaired t-test, α = 0.05.

	Brain region	*P*	Sleep effect^a^
Aspartate	CTX	0.03964	**↓**
cAMP	CBL	0.03137	**↑**
	CTX	0.01661	**↑**
Citicoline	CTX	0.00450	**↓**
Gluconic acid	CBL	0.04422	**↓**
Melanin	CBL	0.00740	**↑**
	MDB	0.01096	**↓**
*N*-Acetyl-L-methionine	MDB	0.02170	**↓**
Ergothioneine	CBL	0.00444	**↓**
Glutathione	CBL	0.04526	**↓**
	HC	0.00656	**↑**
Oxidized glutathione	CBL	0.02988	**↑**
	CTX	0.03764	**↑**
SAM	CBL	0.04349	**↓**
	CTX	0.00361	**↓**
*S*-lactoyl glutathione	CBL	0.01164	**↑**
	HC	0.00717	**↑**
*β*-citryl glutamate	CTX	0.01760	**↓**
Isomer A	CTX	0.00049	**↑**
*N*-acetylaspartate	CTX	0.01524	**↑**
Phenyllactic acid	CTX	0.01685	**↑**
Hydroxyphenyllactic acid	CTX	0.00258	**↑**
Homovanillic acid	CTX	0.00642	**↑**
Adipoyl/methylglutarylcarnitine	CTX	0.01704	**↑**
Decenoylcarnitine	CBL	0.00201	**↑**
	CTX	0.00207	**↑**
	HC	0.04891	**↑**
Hydroxydecanoylcarnitine	CBL	0.00595	**↑**
	CTX	0.00439	**↑**
Hydroxydodecanoylcarnitine	CBL	0.00449	**↑**
	CTX	0.00325	**↑**
	HC	0.02416	**↑**
Hydroxyhexanoylcarnitine	HC	0.00313	**↑**
Hydroxytetradecadiencarnitine	CBL	0.00781	**↑**
	CTX	0.00180	**↑**
Succinylcarnitine	CBL	0.00935	**↓**
Tetradecadiencarnitine	CBL	0.00006	**↑**
	CTX	0.00073	**↑**
Hydroxyoctadecanoic acid	CBL	0.00031	**↑**
	CTX	0.00084	**↑**

^a^Upward arrows indicate higher metabolite levels during sleep-state, downward arrows indicate lower metabolite levels during the sleep-state.

### Cerebellar differences of the gut microbiome-derived metabolite ergothioneine

**Figure 2.**
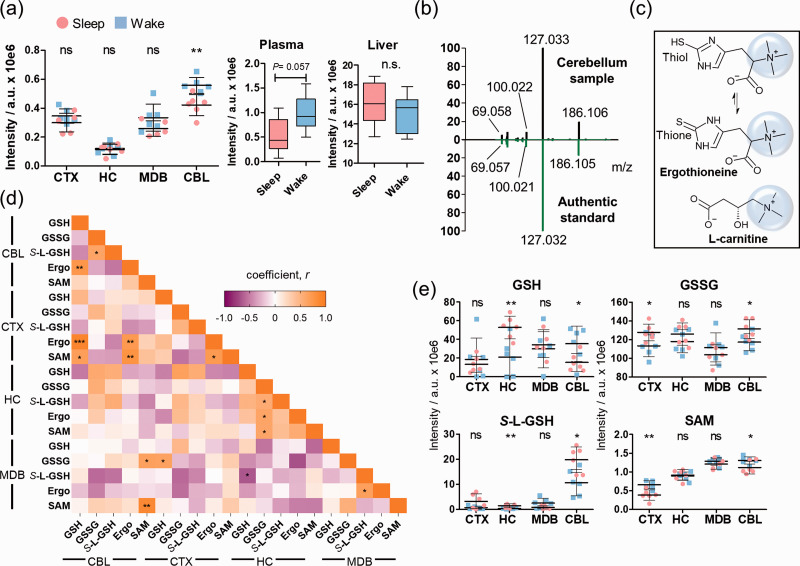
Brain regional effects of sleep on the microbiome-derived metabolite ergothioneine. (a) Mass spectrometric intensities of ergothioneine ([M + K]^+^) in the investigated brain regions, plasma and liver ([M + H]^+^) (N = 6); (b) Structure validation of ergothioneine by comparison of tandem spectra collected from cerebellum (black) and an authentic standard (green); (c) Chemical structures of ergothioneine and carnitine; (d) Heatmap of the correlation coefficients between ergothioneine and other sulfur containing molecules; (e) Distribution of *S*-L-GSH, GSH, GSSG, and SAM in the brain regions (N = 6). Error bars: standard deviation (SD). Two-tailed unpaired t-test: *P < 0.05, **P < 0.01, ***P < 0.001. CBL: cerebellum; CTX: cortex; HC: hippocampus; MDB: midbrain; Ergo: ergothioneine, GSH: glutathione; GSSG: oxidized glutathione; *S*-L-GSH: *S*-lactoyl-glutathione; SAM; *S*-adenosyl-methionine.

The microbiome-derived metabolite ergothioneine,^26^ a metabolite specifically produced by the gut microbiome, was identified to be significantly increased during wakefulness in the cerebellum (Figure 2(a)). Ergothioneine also exhibited higher levels in the plasma of the wake-state group mice compared to the sleep-state, which is expected as it is transported from the gut to the brain via the blood.^27–30^ However, no significant differences between the two groups were detected for ergothioneine in the liver, suggesting that the enrichment of ergothioneine in the brain is due to specific uptake. The chemical structure of ergothioneine was validated using an authentic standard through co-injection experiments and by comparison of the MS/MS spectra in CBL (Figure 2(b)). Ergothioneine contains a thiol moiety in equilibrium with the corresponding thione form that has been attributed to antioxidant properties (Figure 2(c)).^31^ The trimethylammonium moiety is common with carnitine and microbiome-derived metabolites.^32^ Due to the metabolic importance of sulfur containing metabolites, e.g. glutathione and *S*-adenosylmethionine (SAM), that are associated with diverse metabolic reactions including oxidation and methyl group transfer, a regional correlation analysis was performed (Pearson’s coefficient *r*) (Figure 2(d), Supplementary Figure 10, Supplementary Table 4). Each investigated brain area displayed a distinct correlation pattern. Glutathione was negatively correlated in CBL with oxidized glutathione and the pyruvate metabolism intermediate *S*-lactoylglutathione (*S*-L-GSH) but positively in HC. Ergothioneine and glutathione demonstrated a similar sleep-induced pattern in CBL. The opposite was observed in CBL between ergothioneine and both glutathione-analogues (Figure 2(e)).

### Cerebral cortex elevation of an unknown isomer of *β*-citryl-glutamate (1) during sleep

**Figure 3.**
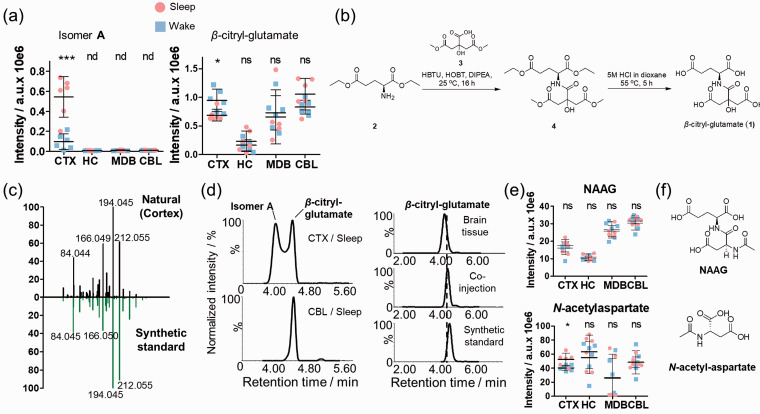
Cerebral cortical elevation of *β*-citryl glutamate isomer **A** during sleep. (a) UPLC-MS intensities of the three examined chromatographic peaks (*m/z* 322.077, [M + H]^+^) in the investigated brain regions during the sleep and the awake state (N = 6); (b) Chemical synthesis of *β*-citryl-glutamate (**1**); (c) *β*-Citryl glutamate (**1**) was validated by MS/MS fragmentation of a cortex sample (black) and the synthesized standard (green); (d) Extracted ion chromatograms of *β*-citryl-glutamate and isomer **A** in the sleep-state in the cortex, the cerebellum and co-injection experiments of synthetic and natural *β*-citryl-glutamate; (e) Distribution of NAAG and *N*-acetylaspartate in the investigated brain regions; (f) Chemical structure of NAAG and *N*-acetyl-aspartate; Error bars: standard deviation (SD). Two-tailed unpaired *t*-test: *P < 0.05, **P < 0.01, ***P < 0.001. CBL: cerebellum; CTX: cortex; HC: hippocampus; MDB: midbrain; NAAG: *N*-acetyl-aspartyl-glutamate.

One significantly increased metabolite during sleep was specific for CTX where it was also present at the highest concentration (Figure 3(a)). The structure was initially assigned to pseudopeptide *β*-citryl-glutamate (**1**) via MS/MS fragmentation comparison with databases. As a second isomer was identified that was reversely altered in CTX and with the identical MS/MS fragmentation pattern, we chemically synthesized *β*-citryl-glutamate (**1**) as it is not commercially available (Figure 3(b), Supplementary Figures 11-12). The straightforward synthesis yielded this compound in highest purity and was used for UPLC-MS/MS co-injection experiments. To our surprise, the major peak at 4.65 min that is present in all tissue samples was identical with the synthetic *β*-citryl-glutamate (**1**) (Figure 3(c) to (d)). Regioisomer **A** at 4.30 min has the exact identical MS/MS spectra and its structure needs to be elucidated (Supplementary Figure 13). No isomer of *β*-citryl-glutamate has been reported before and the presence solely in the cortex with increased concentrations during sleep suggests a regulatory function. *β*-Citryl-glutamate (**1**) is structurally similar to the most abundant dipeptide of the CNS, *N*-acetyl-aspartyl-glutamate (NAAG/Figure 3(e) to (f), Supplementary Figure 14, Supplementary Table 5). NAAG displayed a similar brain distribution to **1** in contrast to isomer **A**, while it was not significantly affected by the sleep/awake state. Furthermore, the amino acid derivative *N*-acetylaspartate displayed CTX-specific elevation during sleep (Figure 3(e) to (f), Supplementary Figure 14, Supplementary Table 5). It is highly abundant in the brain and can serve as a precursor of NAAG.^33^ These results suggest a distinct functional role of isomer **A** in the cortex.

### Sleep-specific alterations of phenylalanine and tyrosine metabolism in the cerebral cortex

**Figure 4.**
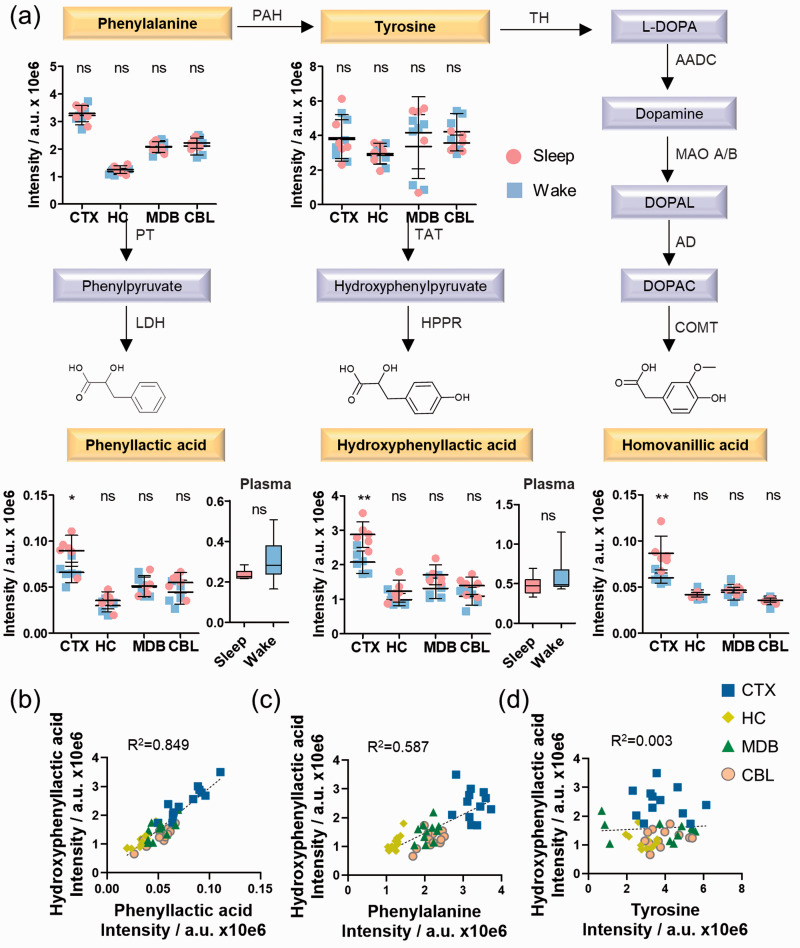
Sleep-induced cortical alterations in phenylalanine and tyrosine metabolism. (a) UPLC-MS intensities of phenylalanine, tyrosine, phenyllactic acid, hydroxyphenyllactic acid, and homovanillic acid in the investigated brain regions for the day/night sample sets (N = 6) as well as phenyllactic acid and hydroxyphenyllactic acid in plasma (N = 6); (b) Similarity analysis of phenyllactic acid and hydroxyphenyllactic acid; (c) Similarity analysis of phenylalanine and hydroxyphenyllactic acid; (d) Similarity analysis of tyrosine and hydroxyphenyllactic acid; (b–d) Analysis in each brain region (N = 6). Error bars: standard deviation (SD); Two-tailed unpaired *t*-test: *P < 0.05, **P < 0.01, ***P < 0.001. AADC: aromatic L-amino acid decarboxylase; AD: aldehyde dehydrogenase; COMT: catechol-*O*-methyl transferase; HPPR: hydroxyphenylpyruvate reductase; LDH: lactate dehydrogenase; MAO: mono-amino oxidase; PAH: phenylalanine hydroxylase; PT: phenylalanine transaminase; TAT: tyrosine aminotransferase; TH: tyrosine hydroxylase.

Phenyllactic acid and hydroxyphenyllactic acid are metabolic downstream products of phenylalanine and tyrosine metabolism. These two compounds were significantly elevated during sleep in the cerebral cortex, while phenylalanine and tyrosine were unaltered (Figure 4(a), Supplementary Figures 15 and 16, Supplementary Table 6). Importantly, no significant differences were observed in the plasma samples. Similar trends for homovanillic acid were observed in CTX, which is the final metabolite of dopamine metabolism. All three carboxylic acid-containing metabolites were distributed similarly with the highest levels in CTX. No correlation was observed for tyrosine, while the regional distribution was significantly similar for phenyllactic acid and hydroxyphenyllactic acid as well as for phenylalanine and hydroxyphenyllactic acid (Figure 4(b)). The observed differences between both arousal states were selective in the cortex for this pathway and have not been described previously.

### Carnitine shuttle during sleep

**Figure 5.**
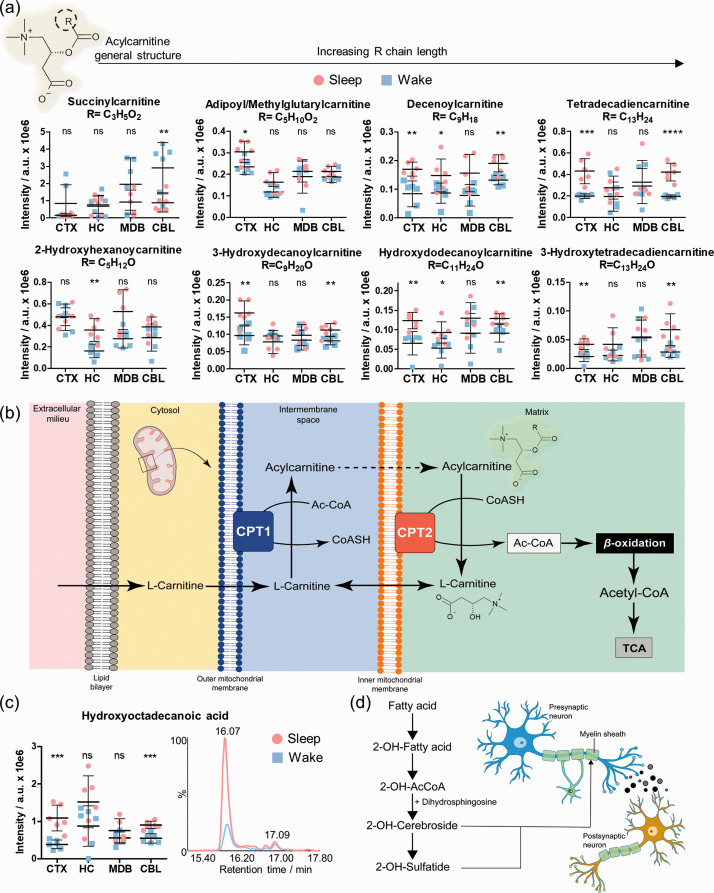
Brain region specific effects of sleep on acylcarnitines. (a) Distribution and sleep/wake differences of selected acylcarnitine conjugates in the investigated brain regions (N = 6 in each group); (b) Simplified schematic illustration of the carnitine shuttle; (c) Distribution of hydroxyoctadecanoic acid in the investigated brain regions (N = 6) and representative EICs for *m/z* 299.2580 ([M-H]^−^) in CTX; (d) Simplified schematic representation of the 2-hydroxylation of fatty acids and their incorporation in the myelin components 2-OH-cerebrosides and 2-OH-sulfatides. Error bars: standard deviation (SD); Two-tailed unpaired *t*-test: *P < 0.05, **P < 0.01, ***P < 0.001. AcCoA: acyl-coenzyme A; CPT1/2: carnitine palmitoyltransferase 1 and 2.

Another metabolite class that was significantly altered between the sleep and the wake state were acylated L-carnitine analogues. Medium-chain (e.g. 2-hydroxyhexanoylcarnitine adipoyl/methylglutarylcarnitine, decenoylcarnitine and 3-hydroxydecanoylcarnitine) and long-chain acyl-carnitines (e.g. hydroxydodecanoylcarnitine, tetradecadiencarnitine and 3-hydroxytetradecadiencarnitine) were detected at increased levels during sleep (Figure 5(a), Supplementary Figures 17 and 20, Supplementary Table 7). Interestingly, different acylated carnitine conjugates were significantly altered in certain brain regions demonstrating brain region-dependent needs of specific fatty acids, as these compounds are part of the carnitine shuttle and fatty acid oxidation key pathways in sleep-wake metabolism. While most of the acyl carnitines were upregulated during sleep, we identified succinylcarnitine to be significantly downregulated during sleep.

The highly abundant metabolite L-carnitine is involved in the mitochondrial *β*-oxidation of fatty acids (Figure 5(b)). Initially, the conjugation of fatty acids and L-carnitine occurs in the outer mitochondrial membrane and is catalyzed by the enzyme carnitine palmitoyltransferease 1 (CPT1). Subsequently, acylcarnitines enter the inner mitochondrial membrane, where they are hydrolyzed to acyl-CoA and L-carnitine via CPT2. The released fatty acids are broken down through *β*-oxidation and the catabolic products enter the TCA cycle.

Among these carnitine conjugates, we also detected several hydroxylated fatty acid-carnitine conjugates that were significantly increased during sleep. The hydroxylation site in position 2 or 3 was determined via MS/MS fragmentation through identification of specific product ions.^34^ We also investigated their corresponding free hydroxylated fatty acids. One representative example is hydroxyloctanodecanoic acid that was significantly elevated during sleep in CTX and CBL (Figure 5(c), Supplementary Figure 21). We considered the main signal of the specific extracted ion chromatogram. Importantly, 2-hydroxylated fatty acids are known moieties of cerebrosides and sulfatides, i.e. hexosylated ceramides and are substantial components of myelin, the substance surrounding the nerve cell axons (Figure 5(d)). These results demonstrate different needs of fatty acids in each brain region.

### Systemic and hepatic sleep metabolomics

**Figure 6.**
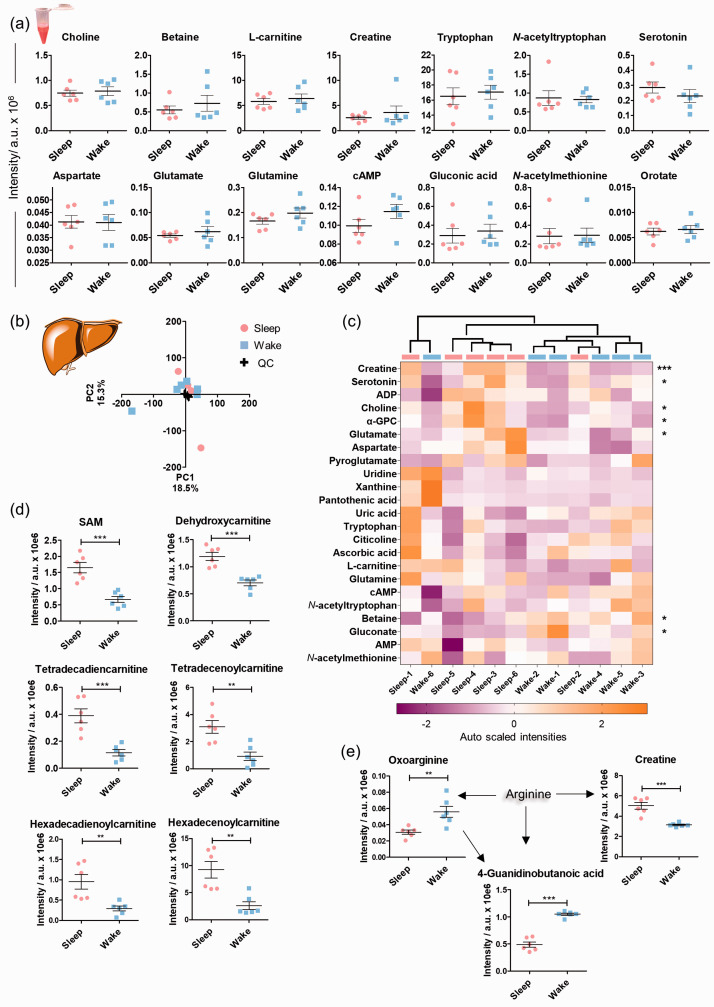
Systemic and hepatic sleep induced metabolic alterations. (a) Plasma sample analysis. UPLC-MS intensities of plasma metabolites illustrate similarities and differences between the day/night sample sets. No statistical significance was observed (two-tailed unpaired t-test); (b) Metabolomics analysis of liver tissue. Scores plot of the principal component analysis on the animals based on the intensities of the included features in both positive and negative MS ionization mode; (c) Heatmap of the identified small metabolites based on their auto scaled intensities (N = 6, features from positive and negative MS ionization mode); (d). Mass spectrometric intensities of significantly modified metabolites in liver tissue in sleep and wake state (N = 6); (e) Significantly altered metabolites of the arginine metabolic pathway; Error bars: standard deviation (SD), two-tailed unpaired *t*-test: *P < 0.05, **P < 0.01, ***P < 0.001.

Plasma and liver samples were also collected from the same animals for control analysis to identify systemic metabolite changes (Figure 6, Supplementary Tables 9–10). No significant systemic alterations were detected for the reported altered brain metabolites *vide supra* (Figure 6(a)). Detailed metabolomics analysis of hepatic tissue samples (Figure 6(b)), however, revealed a stronger effect of sleep on this highly metabolic organ compared to plasma. The liver is a highly circadian-regulated tissue that exhibited significant sleep-specific alterations in several metabolite classes e.g. creatine, serotonin, choline and its metabolites α-glycerophosphocholine (α-GPC) and betaine, glutamate, and gluconate (Figure 6(c)).^35,36^ These metabolites were not found to be altered in the four brain regions. Previously reported circadian regulated metabolites such as SAM and numerous acylcarnitines were also subjected to sleep-specific level changes (Figure 6(d)).^35^ Interestingly, the liver levels of SAM were significantly higher during the sleep state, while the opposite effect was detected in the CTX and CBL. Together with creatine, a number of arginine metabolites displayed sleep induced alterations, not detected in the brain (Figure 6(e), Supplementary Figure 22). Arginine metabolism is highly localized in the hepatic tissue and has also been reported as a “clock-regulated” metabolic pathway.^35,37^ It should be mentioned that *β*-citryl-glutamate and its isomer **A** were neither detected in plasma nor in the liver tissue.

## Discussion

In the present study, metabolic differences between arousal states (sleep vs wake) were explored in four regions of the mouse brain; cortex, hippocampus, midbrain, and cerebellum. This regional brain analysis during sleep provided comprehensive insights into the distinct metabolic changes for the first time compared to previous whole brain analyses. The examined regions were selected to cover different neuroanatomical and functional features of the CNS. Cortical areas are involved in executive and sensorimotor functions and are often a major focus of sleep investigations. The hippocampus constitutes a key region for memory processing and cognition, with memory consolidation as an important process during sleep. The midbrain includes monoaminergic and cholinergic nuclei projecting neurotransmitters and orchestrating neural stimulation for movement. A region considerably neglected in sleep and circadian research is the cerebellum, which plays a crucial role in movement regulation.^38^ Thus, it is evident that each brain region has different metabolic needs and investigation of these regions simultaneously enables the investigation of a wide range of metabolic processes. We identified that the examined brain regions were metabolically altered during sleep. Our analysis demonstrates highly significant alterations in all brain regions depending on the compound class with the majority of changes in the cortex and the cerebellum. In these regions small signaling molecules, such as aspartate and metabolites of the widely studied purinergic pathway, exhibited significant alterations.^22^ On the contrary, the midbrain includes the circadian rhythm-related hypothalamic nuclei, i.e. the suprachiasmatic nucleus of the hypothalamus, for which the lowest extent of sleep-specific metabolic alterations was observed potentially due to the lack of receptors for endogenous signals.^39^ We decided to investigate the hypothalamus as part of the midbrain as dissection was not feasible in mice due to their small size. Importantly, our systemic investigation of plasma and liver samples demonstrated specific localized difference in the brain for most of metabolites described in this study. Furthermore, the investigation of hepatic tissue identified members of the arginine pathway altered as previously reported.

The gut microbiome derived metabolite ergothioneine was significantly reduced in the cerebellum during sleep. Ergothioneine is produced by the bacteria of the species *Lactobacillus* in the gut, which have also been reported to be under circadian regulation.^26,40^ This metabolite is actively transported into neurons via the organic cation/carnitine transporter 1 (OCTN1), which is highly expressed in the cerebellum where this metabolite has a stimulating effect.^28,41^ The observed significantly reduced levels in the cerebellum during sleep could be due to a reduced neuronal cerebellar activity or sleep-induced alterations of the gut-brain axis, which suggests a functional role. The statistically significant increased levels during wakefulness could relate to its antioxidant properties and neuroprotective effects to quench reactive oxygen species (ROS) derived from higher neuronal activity.^26^ This is of particular importance as specific brain functions are known for similar metabolites from the gut-brain axis that are linked to carnitine mediated fatty acid oxidation.^32^ The absence of significant circadian regulation of ergothioneine in the liver further supports the potential functionality of the molecule in the cerebellum, highlighting the importance of the gut-brain axis.

Increased cortical levels of *β*-citryl glutamate have been described in the newborn cortex, leading to the discovery of the molecule in the brain.^42^ Since the discovery, this metabolite and its function in the brain have not been investigated intensely and merely described as a potential implication in depression.^43^ Interestingly, the significantly higher levels of *β*-citryl-glutamate in the brain after birth were related to metabolic shifts from glucose utilization to oxidative processes. The discovery of a previously unknown regioisomer of *β*-citryl-glutamate (isomer **A**), which is specifically upregulated in the cortex during sleep, suggests additional functions and importance of these modified dipeptides in the brain and its development. We have additionally revealed altered quantities in different brain regions for the first time.

We also detected increased levels of phenyllactic acid, hydroxyphenyllactic acid and homovanillic acid specifically in the cortex during sleep. These metabolites are metabolic end products of phenylalanine and tyrosine metabolism. Phenyllactic acid and hydroxyphenyllactic acid were described with antioxidant properties by decreasing ROS production in both mitochondria and neutrophils.^44^ In addition, the phenolic moiety in hydroxyphenyllactic and homovanillic acid has been associated with neurological disorders such as schizophrenia and autism.^45^ Homovanillic acid is the final product of dopamine metabolism and elevated levels of this metabolite may correlate with an increased dopamine turnover.^46^ The formation of homovanillic acid is mediated by the enzyme monoamine oxidase that is a marker for higher oxidative processes.^47^

Shift towards fatty acid metabolism, exemplified by elevated acyl-carnitines and *β*-oxidation, represents an additional important compound class strengthening the findings of an oxidative energetic shift during sleep. It has previously been reported that sleep is associated with a significant decline in cerebral glucose metabolism.^6,7,48,49^ Our results now reveal increased levels of hydroxylated long-chain fatty acids as carnitine conjugates during sleep. This metabolic shift can be attributed to the higher energy yield through fatty acid oxidation compared to carbohydrate metabolism. The *β*-oxidation process is unfavorable for the brain during wakefulness with these higher energy demands due to the generation of high amounts of neurotoxic ROS.^50^ This process compensates for heat and water loss in tissue during sleep, which leads to an electrolyte imbalance.^6^ Regional differences in the altered acyl-carnitines detected can be linked to cellular variations in fatty acid oxidation, with astrocytes as the predominant sites of this reaction. Overall astrocyte densities differ substantially per brain region with the cortex, hippocampus and cerebellum yielding approximate respective densities of 29,500, 14,500 and 1,500 cells/mm^2^.^51^ Therefore, differences in the cell density among the examined regions can reflect distinct metabolic requirements with respect to fatty acids of different oxidation states and chain lengths.

Although acylcarnitines have been strongly associated with the *β*-oxidation of fatty acids, their roles and functions in the brain exceed beyond this catabolic reaction.^52^ Acylcarnitines have also been established as biomarkers of mitochondrial function and important factors in ketosis. Moreover, this compound class is involved in neuroprotection and enhancement of the cholinergic function and synthesis of acetylcholine.^53^ Long-chain acylcarnitines, such as palmitoylcarnitine, can participate in the synthesis of complex lipids related to neural membranes and signal transduction. In addition, this increase in lipid content can be related to higher myelination during sleep, a process required for the maintenance of neural connections. Genome-wide profiling of oligodendrocytes after sleep demonstrated increased expression of genes involved in the promotion of oligodendrocyte precursor cell proliferation, lipid synthesis and myelination.^54^ This is further supported by increased levels of hydroxylated acylcarnitines in our study. These 2-hydroxylated fatty acids are major components of the sphingolipids cerebrosides and sulfatides, which form the myelin sheath of the neural axons.^55^

In conclusion, our results provide for the first time detailed metabolic profiles in four major brain regions during the sleep and the wake states of the mouse brain. The discovery of diverse compound classes that are linked to oxidative stress, energy metabolism changes and gut-brain axis derived metabolites during the subjective night are new insights into metabolic regulation in the brain. The discovery of an unknown neuropeptide lays the foundation for future mechanistic and metabolic studies to understand brain metabolism and metabolic processes in the circadian rhythm. Our findings lay out the high impact of the circadian rhythm on the brain metabolism and demonstrate that more brain region specific neurochemical alterations exist that cannot be detected through investigation of systemic or hepatic metabolic changes. As the majority of metabolites described belong to metabolic pathways that are highly conserved among species, these findings are of high relevance for human brain metabolism as well.

## Supplemental Material

sj-pdf-1-jcb-10.1177_0271678X211033358 - Supplemental material for Differential regulation of oxidative stress, microbiota-derived, and energy metabolites in the mouse brain during sleep Click here for additional data file. Supplemental material, sj-pdf-1-jcb-10.1177_0271678X211033358 for Differential regulation of oxidative stress, microbiota-derived, and energy metabolites in the mouse brain during sleep by Theodosia Vallianatou, Weifeng Lin, Nicholas B Bèchet, Mario SP Correia, Nagesh C Shanbhag, Iben Lundgaard and Daniel Globisch in Journal of Cerebral Blood Flow & Metabolism
