# Supplementary material for: Differential regulation of oxidative stress, microbiota-derived, and energy metabolites in the mouse brain during sleep
Source: J Cereb Blood Flow Metab. 2021 Jul 22;41(12):3324–38. doi: 10.1177/0271678X211033358 (PMC8669215; doi:10.1177/0271678X211033358)
Supplement: sj-pdf-1-jcb-10.1177_0271678X211033358 - Supplemental material for Differential regulation of oxidative stress, microbiota-derived, and energy metabolites in the mouse brain during sleep [file sj-pdf-1-jcb-10.1177_0271678X211033358.pdf]

Supplementary Information

Differential regulation of oxidative stress, microbiota-derived, and energy metabolites in the mouse brain during sleep

Theodosia Vallianatou, Weifeng Lin, Nicholas B. Bèchet, Mario S.P. Correia, Nagesh C. Shanbhag, Iben Lundgaard, Daniel Globisch

Daniel Globisch, Email: daniel.globisch@scilifelab.uu.se

This PDF file includes:

Figures S1 to S22
Tables S1 to S10
SI References

Supplementary Figures

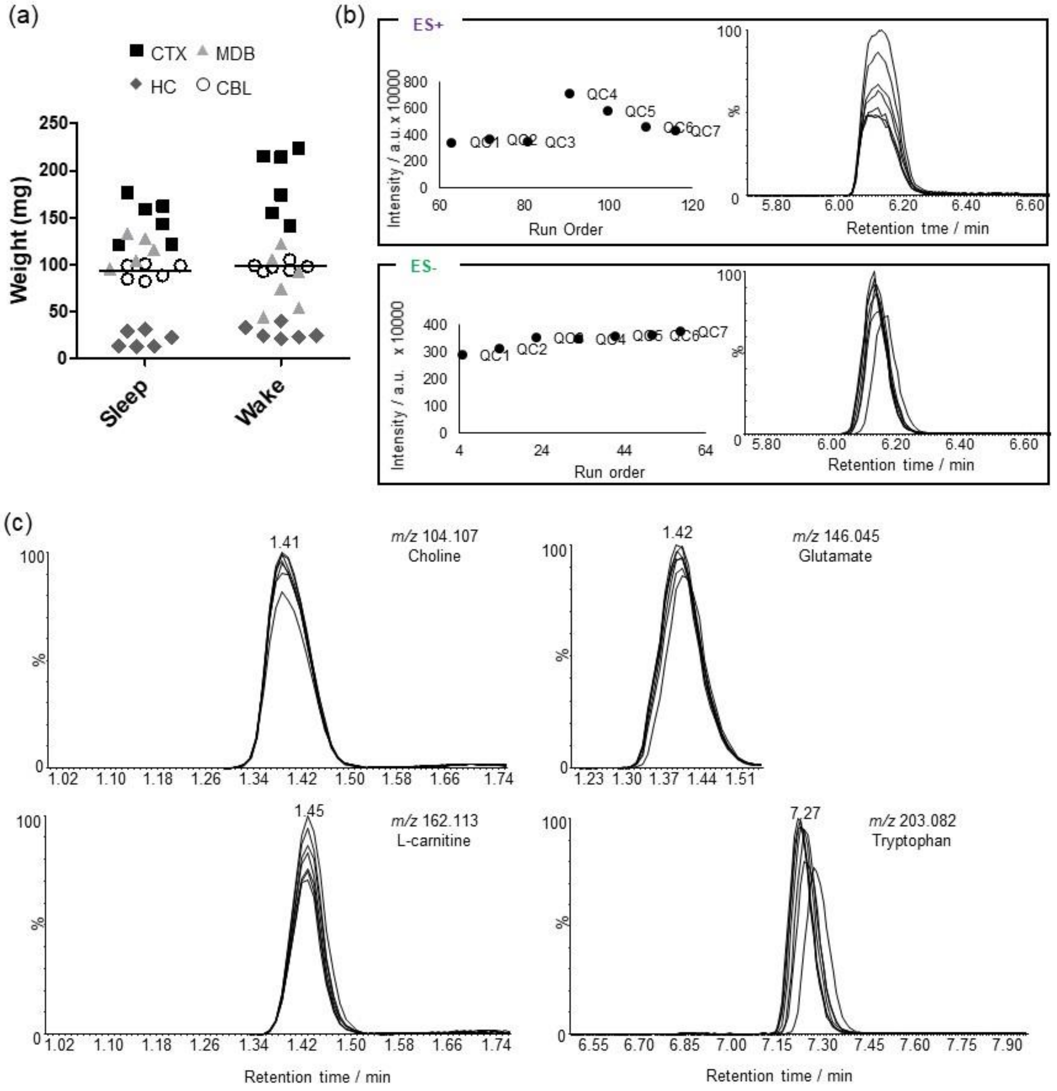

Supplementary Figure 1. Quality control for the metabolomics analysis. (a) Distribution of the brain regions weight (mg) in sleep-state and wake-state animals. (b) . Internal standard stability. UPLC-MS analysis time (run order) of C-13 isotopically labeled phenylalanine in positive (ES+, *m/z* 176.113) and negative (ES-, *m/z* 174.098) ionization mode in the quality control (QC) samples. (c) Representative UPLC-MS chromatograms of endogenous brain metabolites in the quality control samples. Choline and L-carnitine were detected in positive ionization mode, glutamate and tryptophan were detected in negative ionization mode and demonstrate high stability

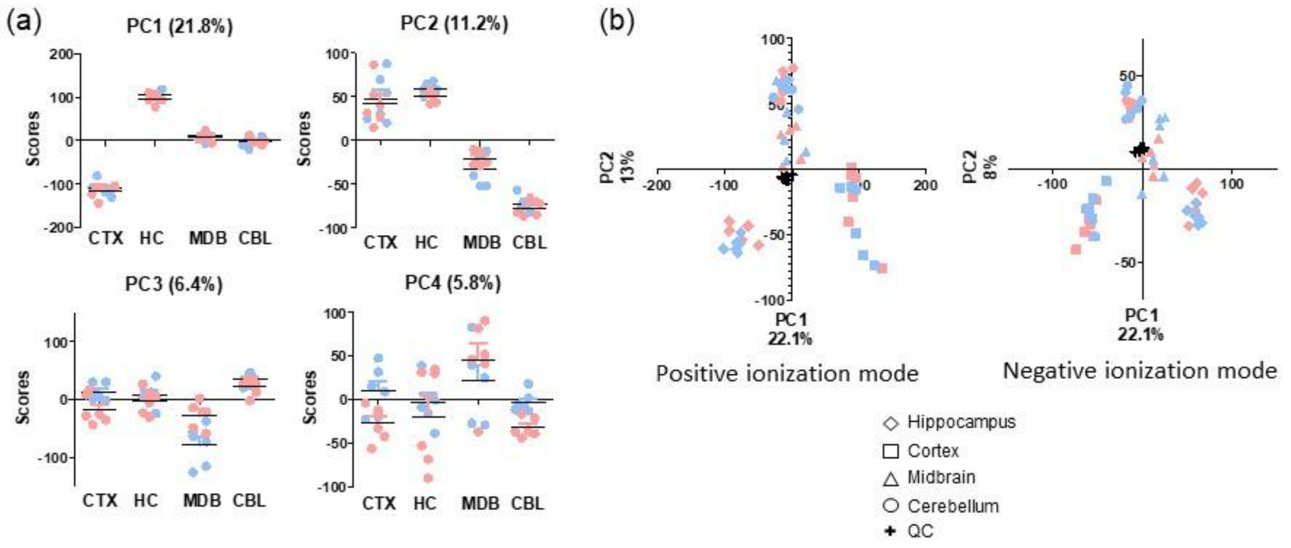

Supplementary Figure 2. Principal component analysis of the global UPLC-MS metabolomics data. (a) Score values of the four principal components of the combined positive and negative ionization mode UPLC-MS data presented as separate dot plots to highlight the main variance in each component. (b) Principal component analysis in positive and negative ionization mode separately. Abbreviations: CBL, cerebellum; CTX, cortex; HC, hippocampus; MDB, midbrain.

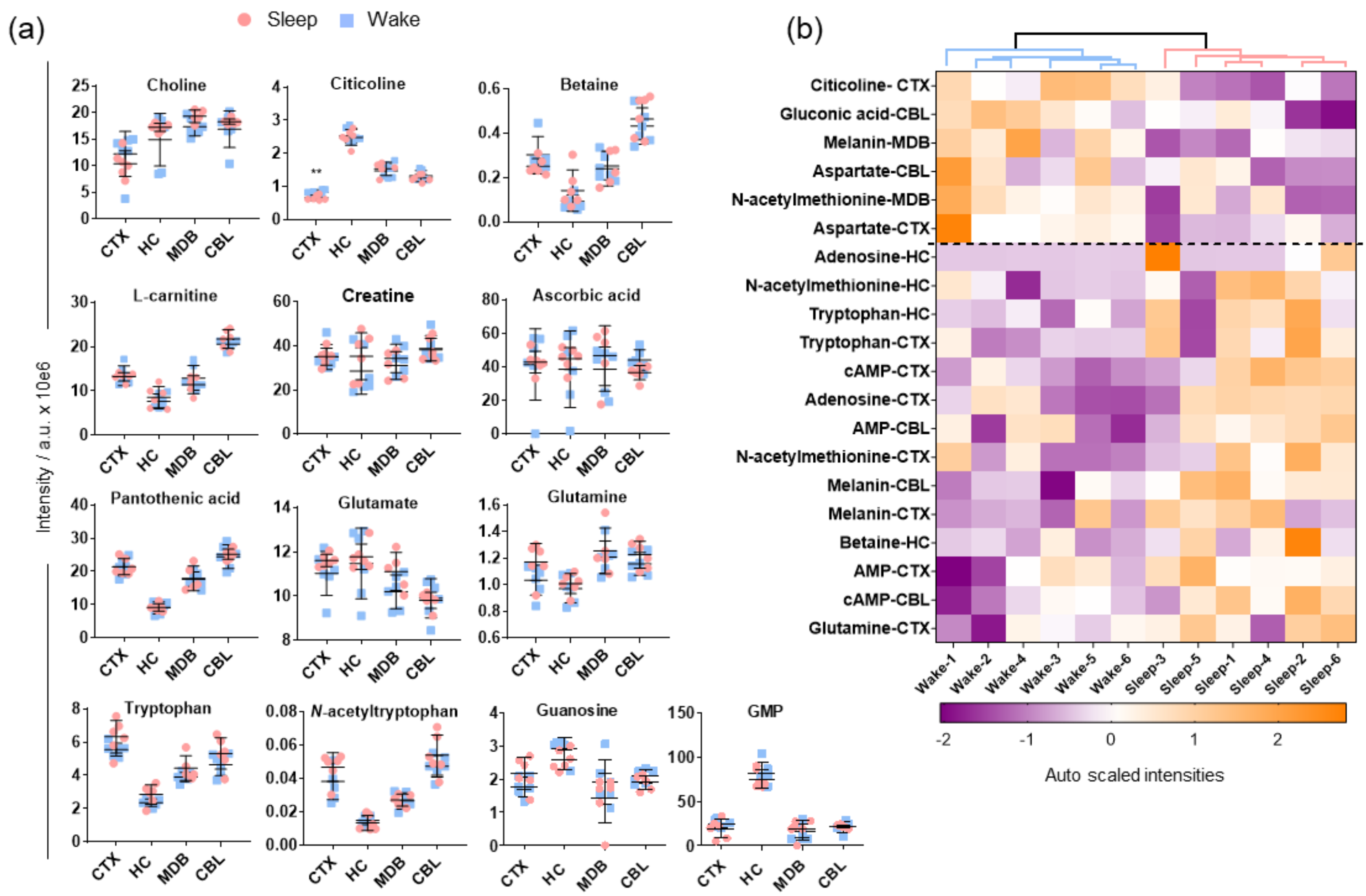

Supplementary Figure 3. Brain regional differences of selected brain molecules. (a) Dot plots of selected brain metabolites and neurotransmitters in both groups and all examined regions, ordered in an anatomical orientation from the most anterior part to the cerebellum; (b) Heatmap of the top twenty molecules (t-test) based on their auto scaled intensities (N=6). Error bars represent the standard deviation (SD); Two tailed unpaired *t*-test: $^{**}P < 0.01$. Abbreviations: CBL, cerebellum; CTX, cortex; HC, hippocampus; MDB, midbrain

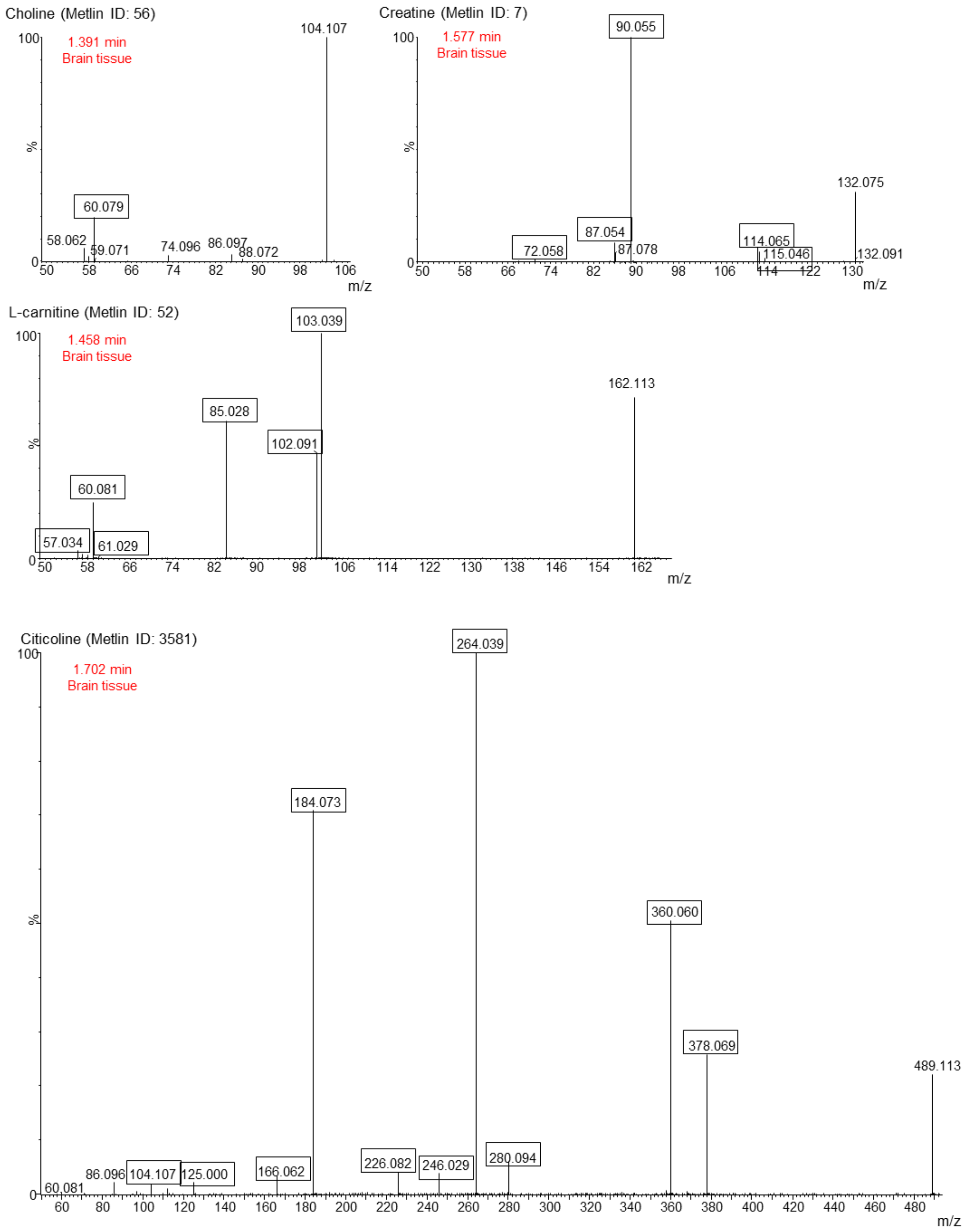

Supplementary Figure 4. Tandem MS/MS spectra for the structural validation of brain metabolites in positive ionization mode. Pooled brain tissue was used for the UPLC-MS/MS analysis and spectra acquired at 20 eV. MS/MS spectra were compared to experimental MS/MS spectra available in the Metlin database and common product ions are annotated with a black rectangle. Metlin ID numbers are provided.

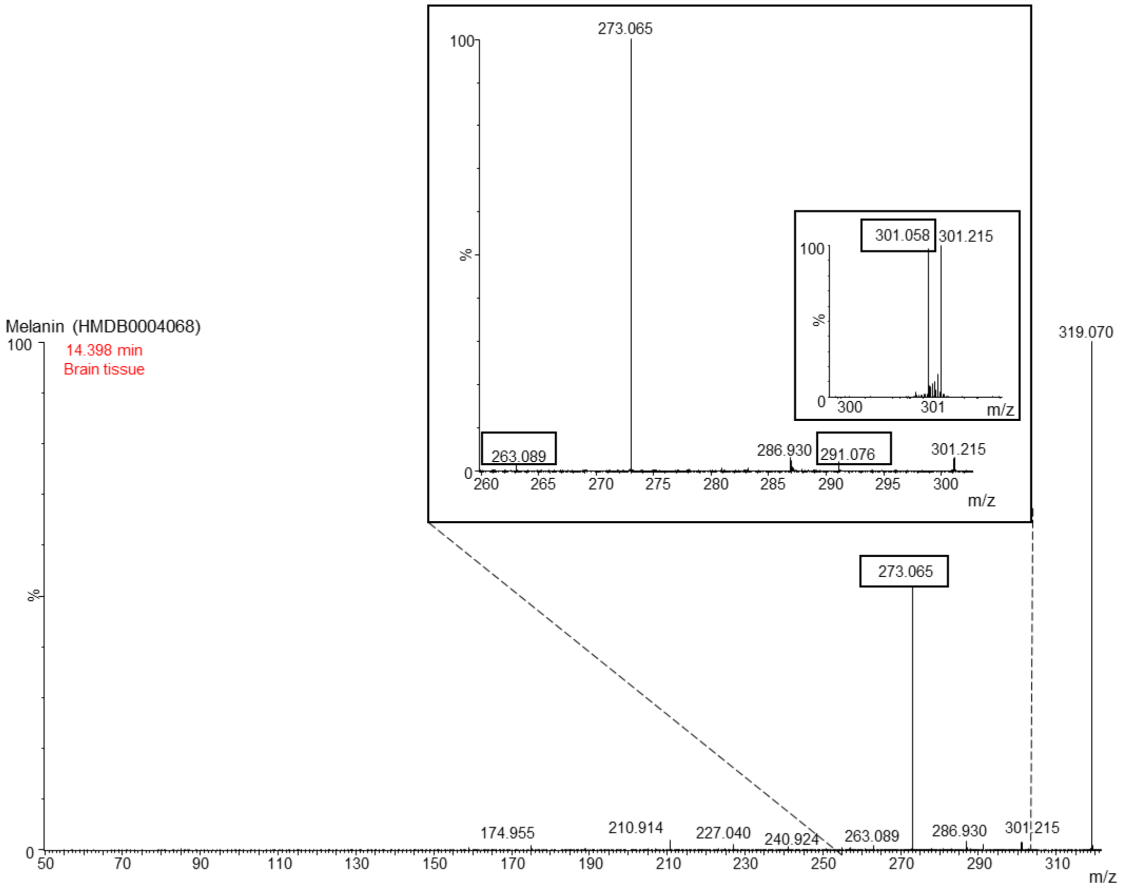

Supplementary Figure 5. Tandem MS/MS spectra for the structural validation of melanin in positive ionization mode. Pooled brain tissue was used for the UPLC-MS/MS analysis and spectra acquired at 10 eV. MS/MS spectra were compared to predicted MS/MS spectra available in the HMDB database and common product ions are annotated with a black rectangle. HMDB ID number is provided.

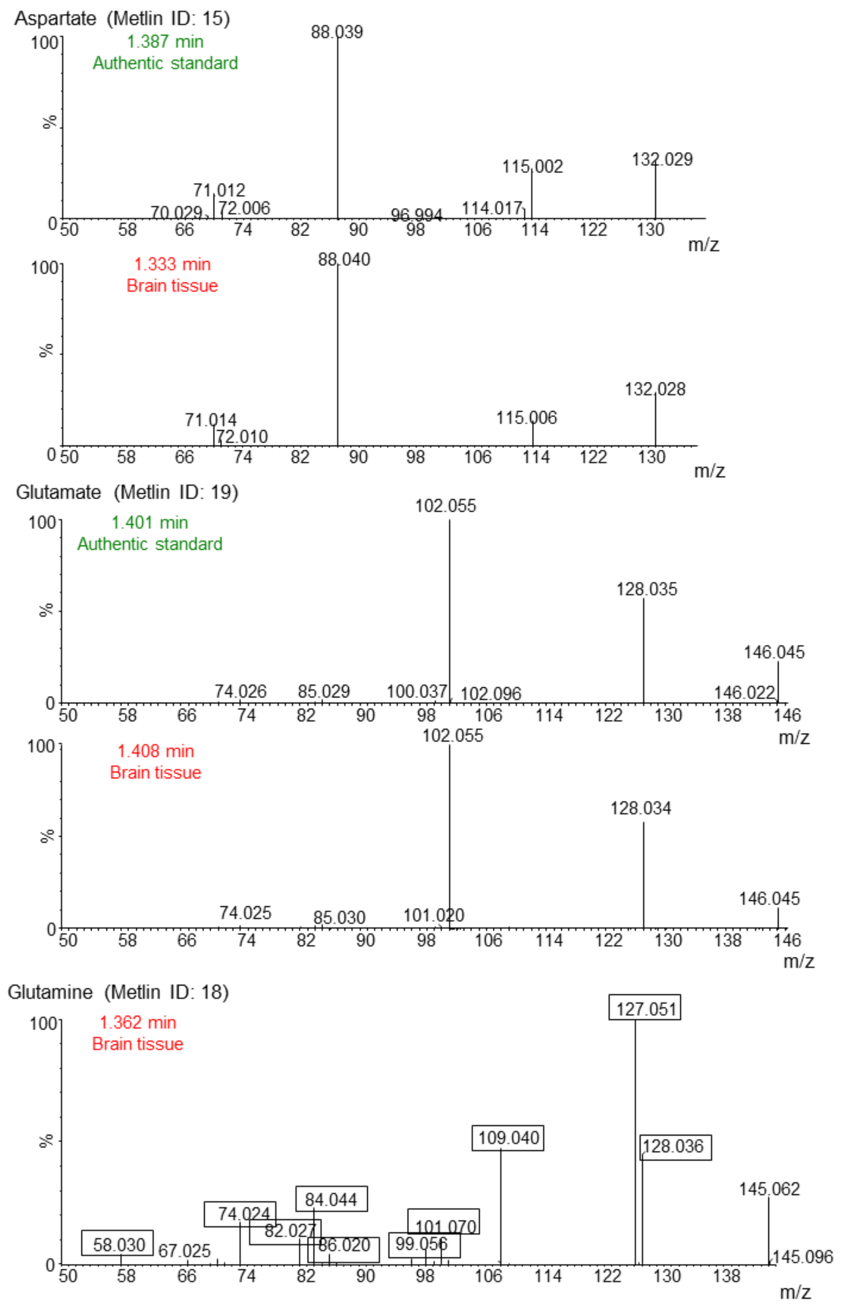

Supplementary Figure 6. Tandem MS/MS spectra for the structural validation of brain metabolites in negative ionization mode. Pooled brain tissue was used for the UPLC-MS/MS analysis and spectra acquired at 20 eV. In the case of aspartate and glutamate the acquired MS/MS spectra were compared to MS/MS spectra collected in authentic standards. In the case of glutamine, MS/MS spectra were compared to experimental MS/MS spectra available in the Metlin database and common product ions are annotated with a black rectangle. Metlin ID numbers are provided.

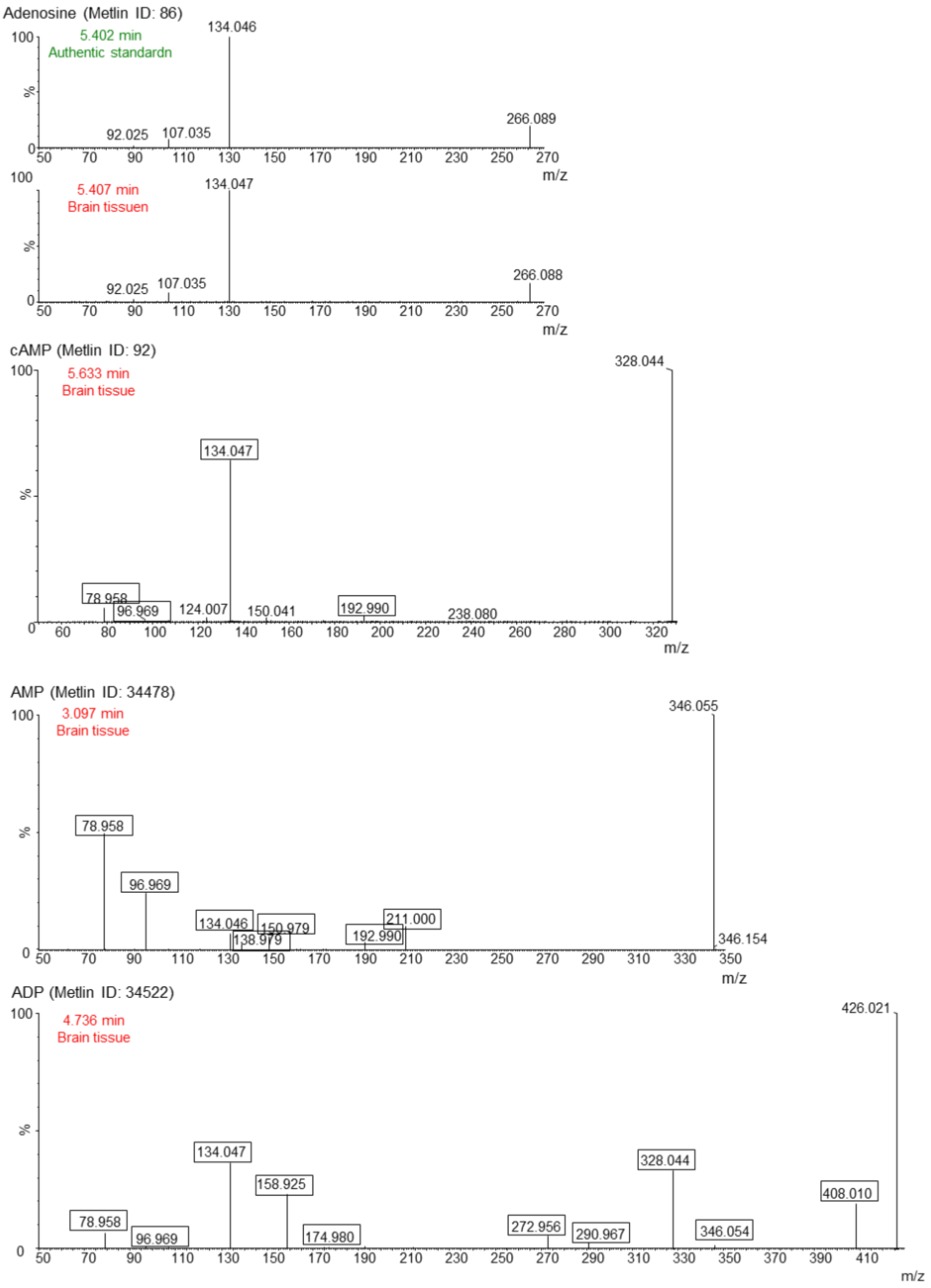

Supplementary Figure 7. Tandem MS/MS spectra for the structural validation of brain metabolites in negative ionization mode. Pooled brain tissue was used for the UPLC-MS/MS analysis and spectra acquired at 20 eV. In the case of adenosine, the acquired MS/MS spectra were compared to MS/MS spectra collected in authentic standards. Otherwise, MS/MS spectra were compared to experimental MS/MS spectra available in the Metlin database and common product ions are annotated with a black rectangle. Metlin ID numbers are provided.

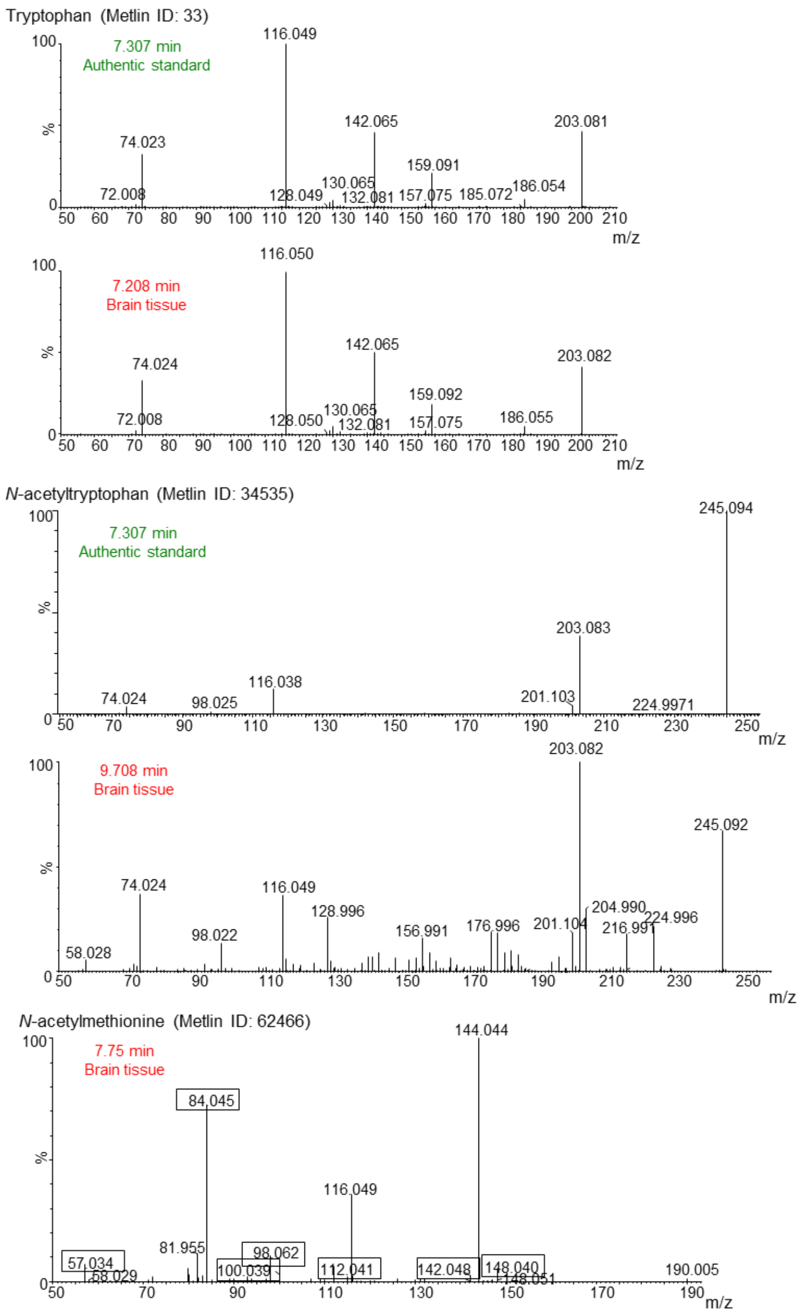

Supplementary Figure 8. Tandem MS/MS spectra for the structural validation of brain metabolites in negative ionization mode. Pooled brain tissue was used for the UPLC-MS/MS analysis and spectra acquired at 20 eV. In the case of tryptophan and *N*-acteyltryptophan, the acquired MS/MS spectra were compared to MS/MS spectra collected in authentic standards. In the case of *N*-acetylmethionine, MS/MS spectra were compared to experimental MS/MS spectra available in the Metlin database and common product ions are annotated with a black rectangle. Metlin ID numbers are provided.

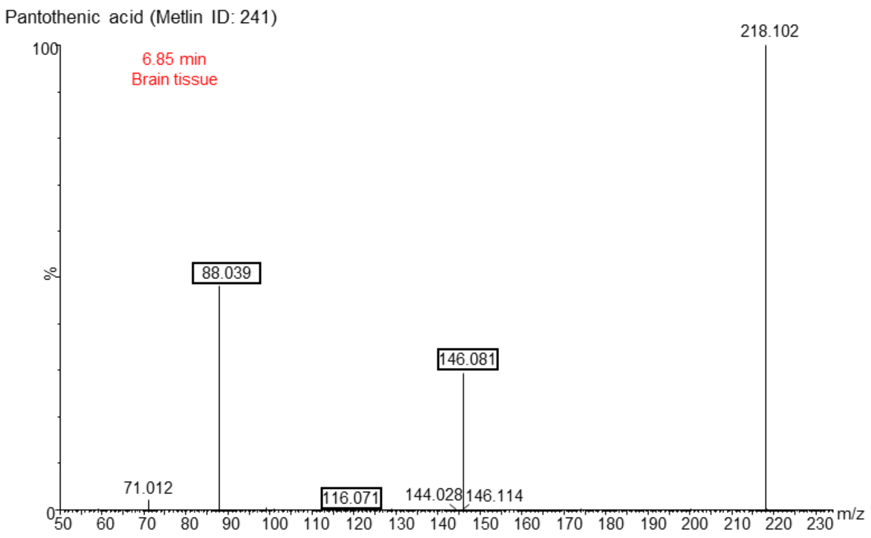

Supplementary Figure 9. Tandem MS/MS spectra for the structural validation of pantothenic acid in negative ionization mode. Pooled brain tissue was used for the UPLC-MS/MS analysis and spectra acquired at 20 eV. MS/MS spectra were compared to experimental MS/MS spectra available in the Metlin database and common product ions are annotated with a black rectangle. Metlin ID number is provided.

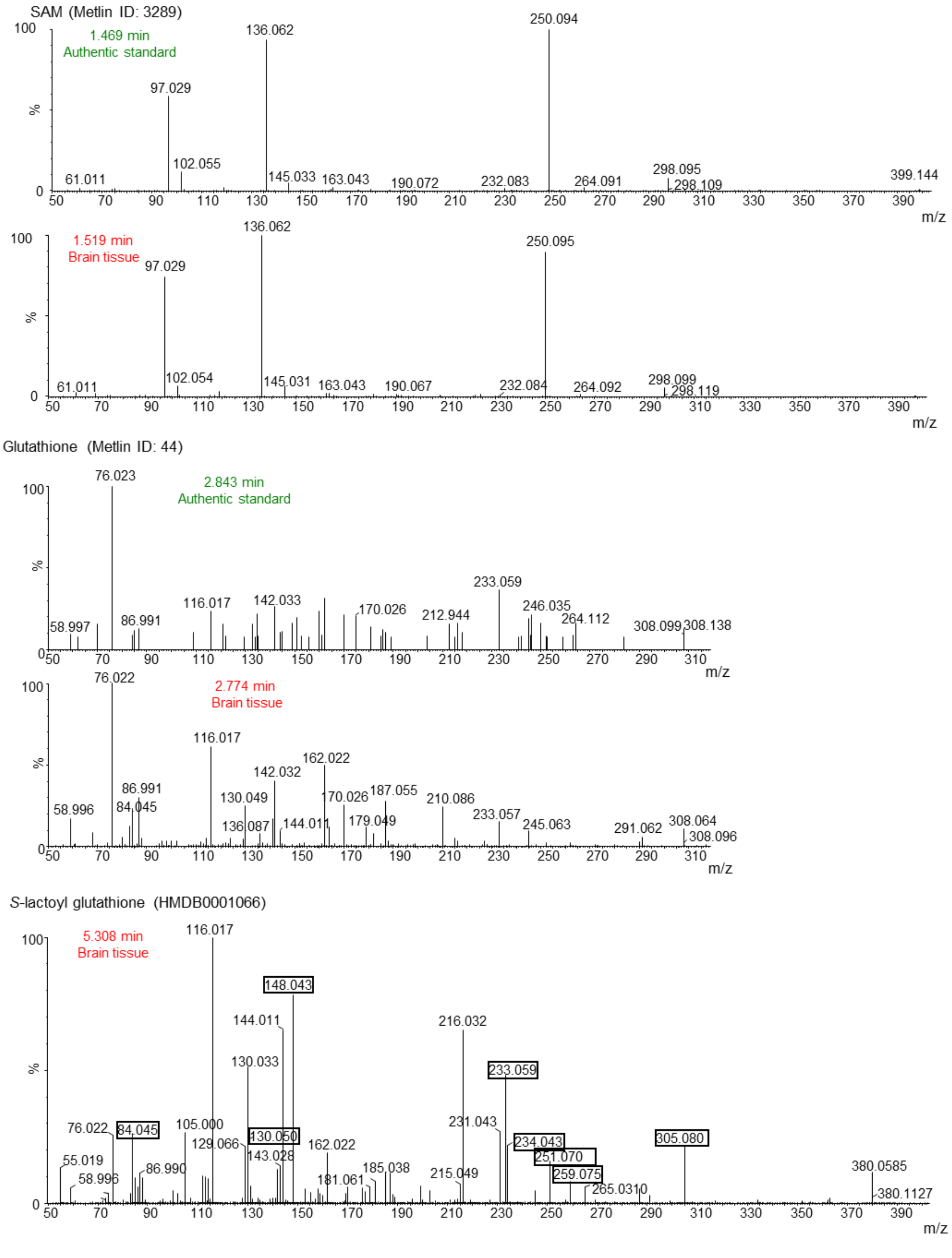
SAM (Metlin ID: 3289)
1.469 min
Authentic standard
136.062
250.094
97.029
102.055
61.011
145.033
163.043
190.072
232.083
264.091
298.095
298.109
399.144
1.519 min
Brain tissue
136.062
250.095
97.029
102.054
61.011
145.031
163.043
190.067
232.084
264.092
298.099
298.119
Glutathione (Metlin ID: 44)
76.023
2.843 min
Authentic standard
233.059
116.017
142.033
170.026
212.944
246.035
264.112
58.997
86.991
308.099
308.138
76.022
2.774 min
Brain tissue
116.017
162.022
142.032
86.991
130.049
170.026
187.055
210.086
233.057
58.996
84.045
136.087
144.011
179.049
245.063
291.062
308.064
308.096
S-lactoyl glutathione (HMDB0001066)
5.308 min
Brain tissue
116.017
148.043
144.011
216.032
130.033
233.059
231.043
76.022
84.045
105.000
130.050
129.066
234.043
305.080
162.022
251.070
55.019
143.028
86.990
58.996
185.038
181.061
215.049
259.075
265.0310
380.0585
380.1127
100
%
0
m/z

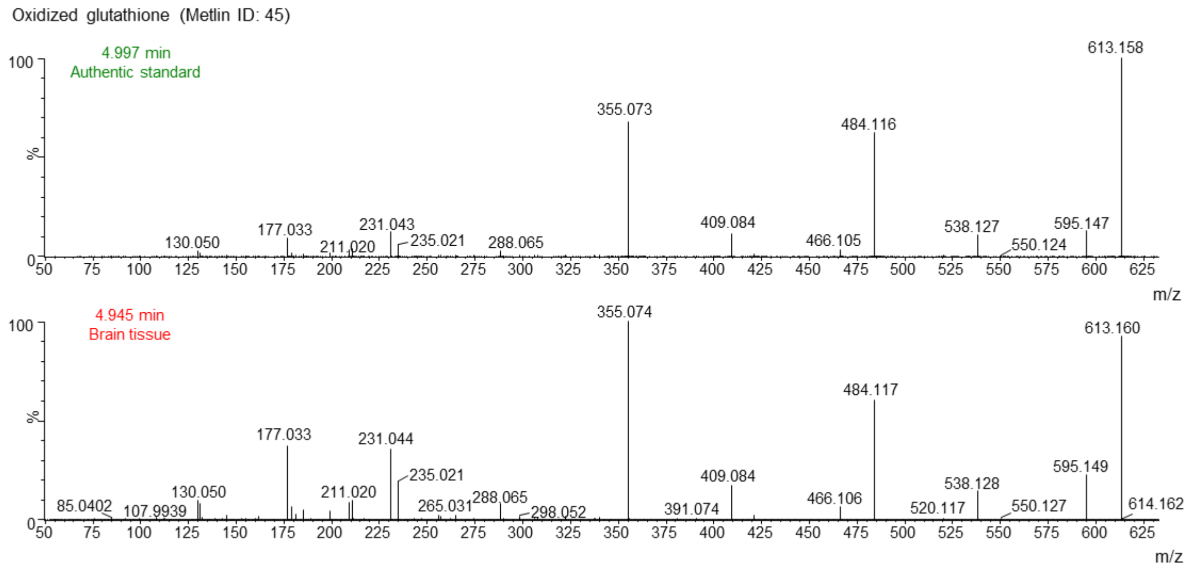

Supplementary Figure 10. Tandem MS/MS spectra for the structural validation of sulfur containing brain metabolites in positive ionization mode. Pooled brain tissue was used for the UPLC-MS/MS analysis and spectra acquired at 20 eV. In the case of SAM, glutathione and oxidized glutathione, the acquired MS/MS spectra were compared to MS/MS spectra collected in authentic standards. In the case of *S*-lactoylglutathione, MS/MS spectra were compared to predicted MS/MS spectra available in the Metlin database and common product ions are annotated with a black rectangle. Metlin ID numbers are provided.

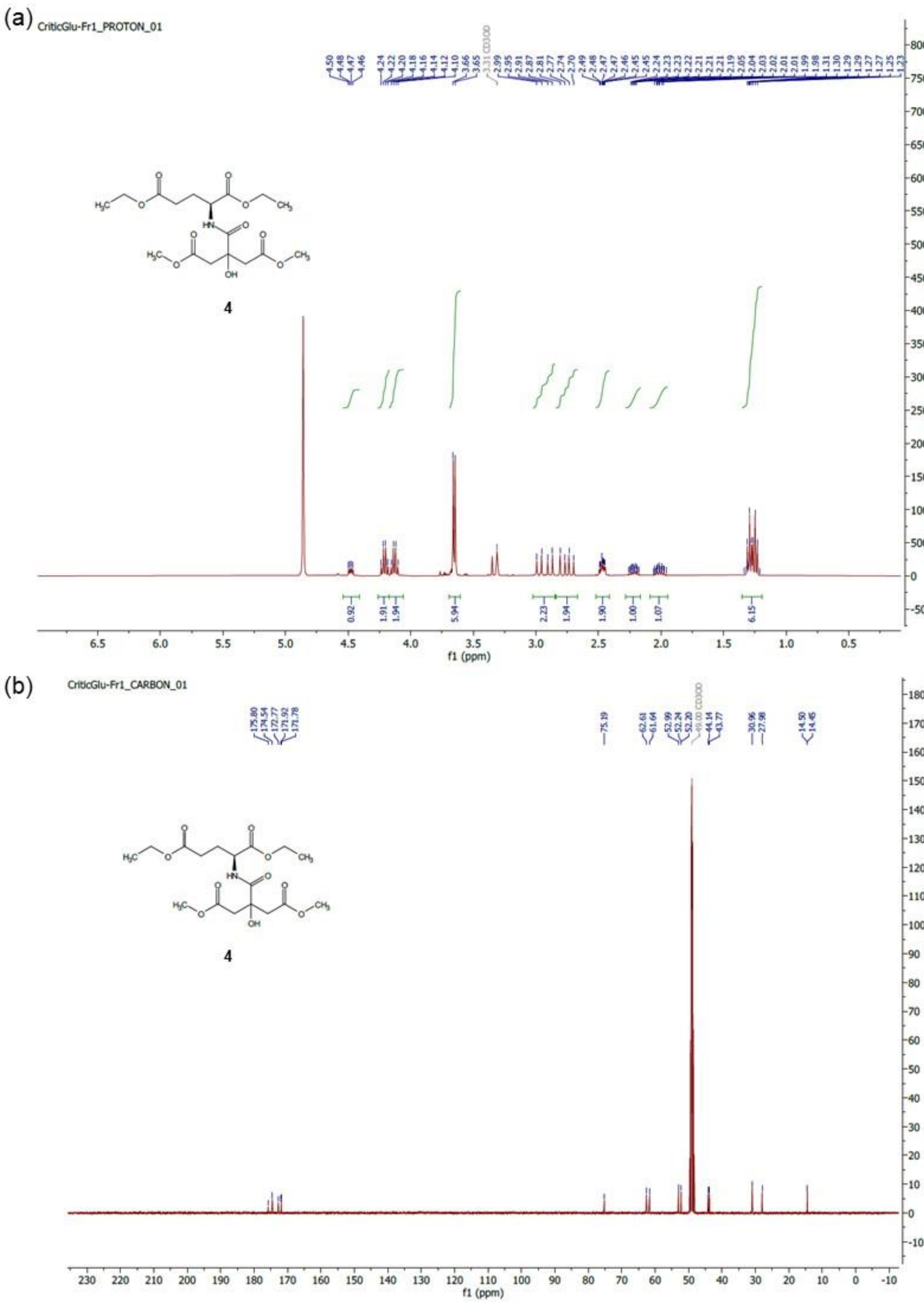

Supplementary Figure 11. NMR spectra for synthetic intermediate 4. (a) ^{1}H-NMR data obtained for compound **4**. (b) ^{13}C NMR data obtained for compound **4**.

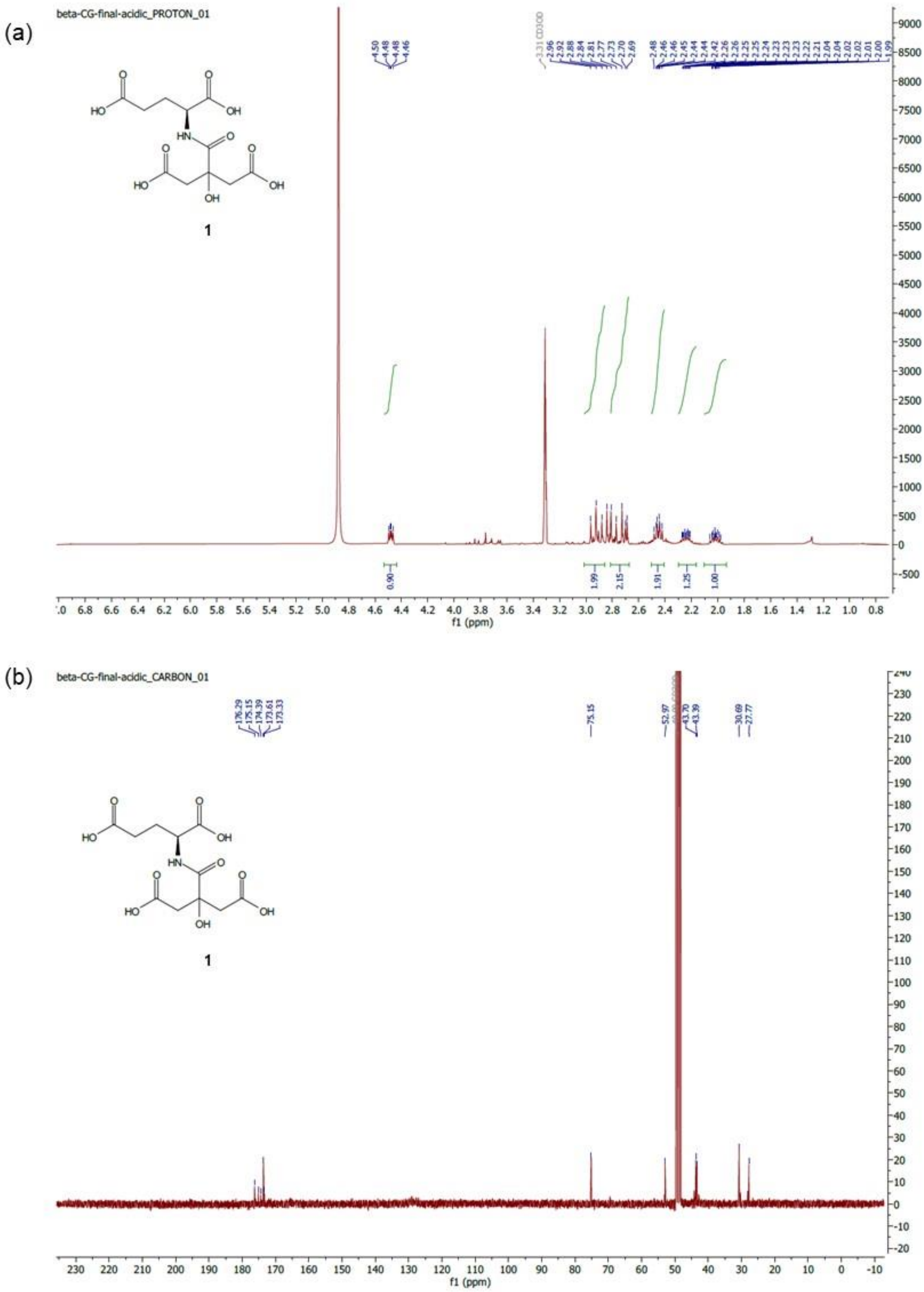

Supplementary Figure 12. NMR spectra for *β*-citryl-glutamate (**1**). (a) ^{1}H-NMR data obtained for compound **1**. (b) ^{13}C NMR data obtained for compound **1**.

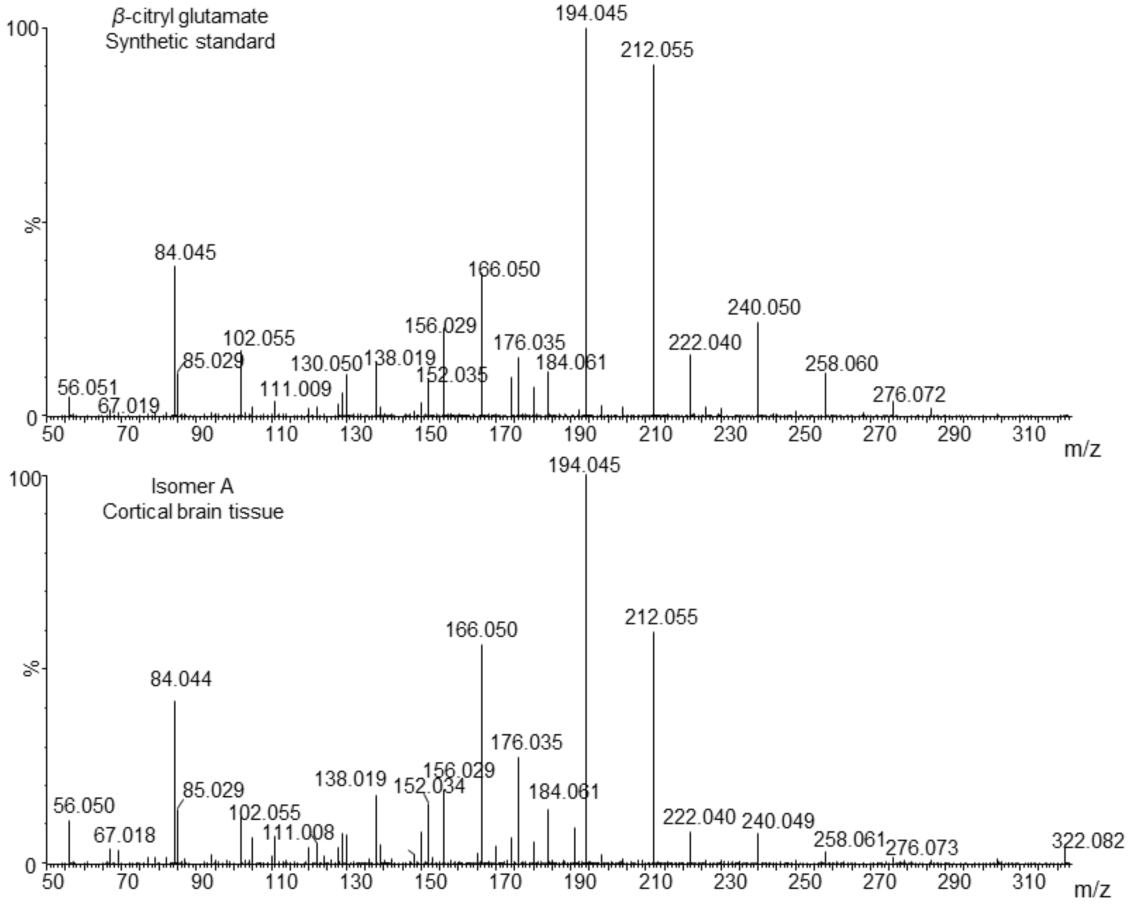

Supplementary Figure 13. Comparison of MS/MS fragmentation spectra of the ***β*****-citryl-glutamate and isomer A.** Pooled cortical brain tissue was used for the UPLC-MS/MS analysis and spectra acquired at 20 eV in negative ionization mode. The MS/MS spectra were compared to the MS/MS spectra acquired for the synthetic standard of *β*-citryl glutamate at 20 eV.

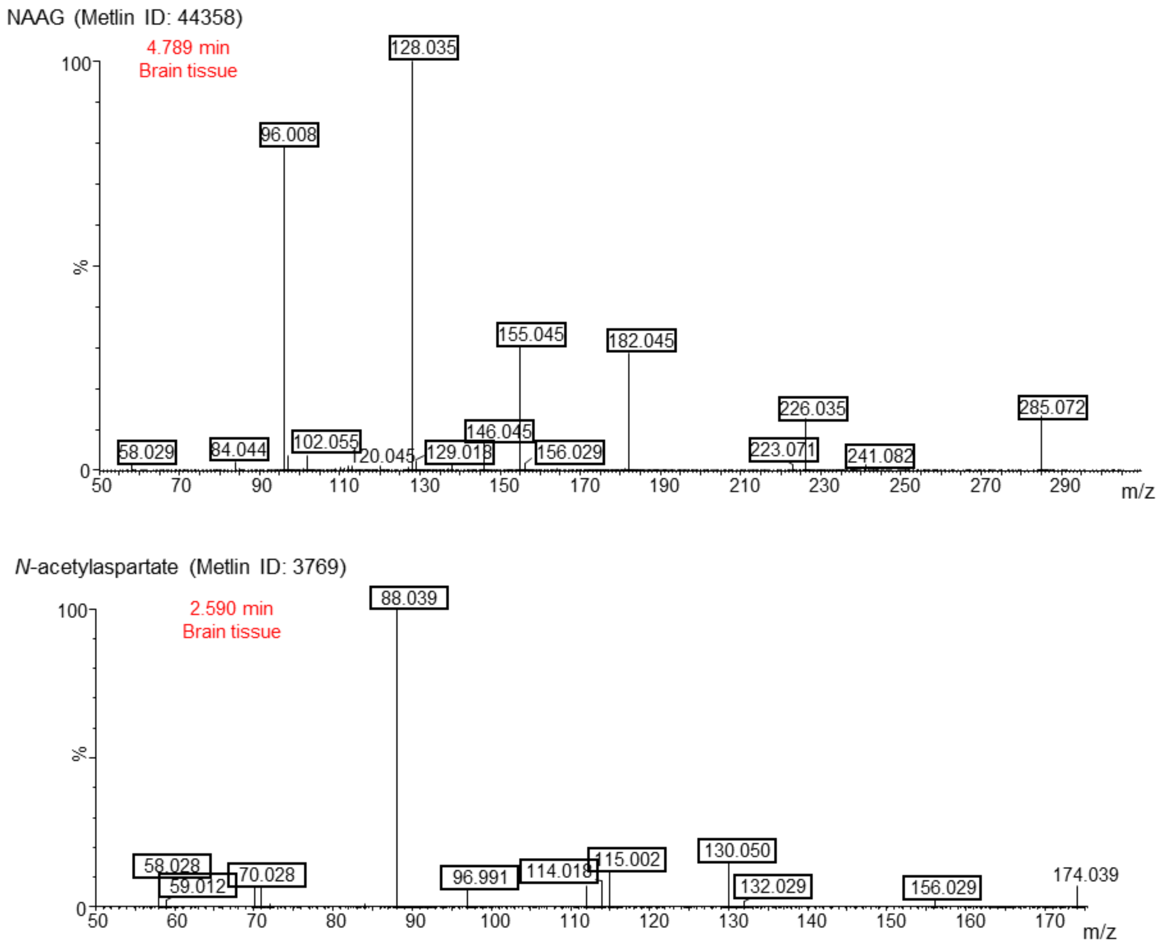

Supplementary Figure 14. Structural validation and brain distribution of NAAG and *N*-acetylaspartate. Tandem MS/MS spectra for the structural validation of NAAG and *N*-acetylaspartate in negative ionization mode. Pooled brain tissue was used for the UPLC-MS/MS analysis and spectra acquired at 20 eV. The acquired MS/MS spectra were compared to experimental MS/MS spectra available in the Metlin database and common product ions are annotated with a black rectangle. Metlin ID numbers are provided. (

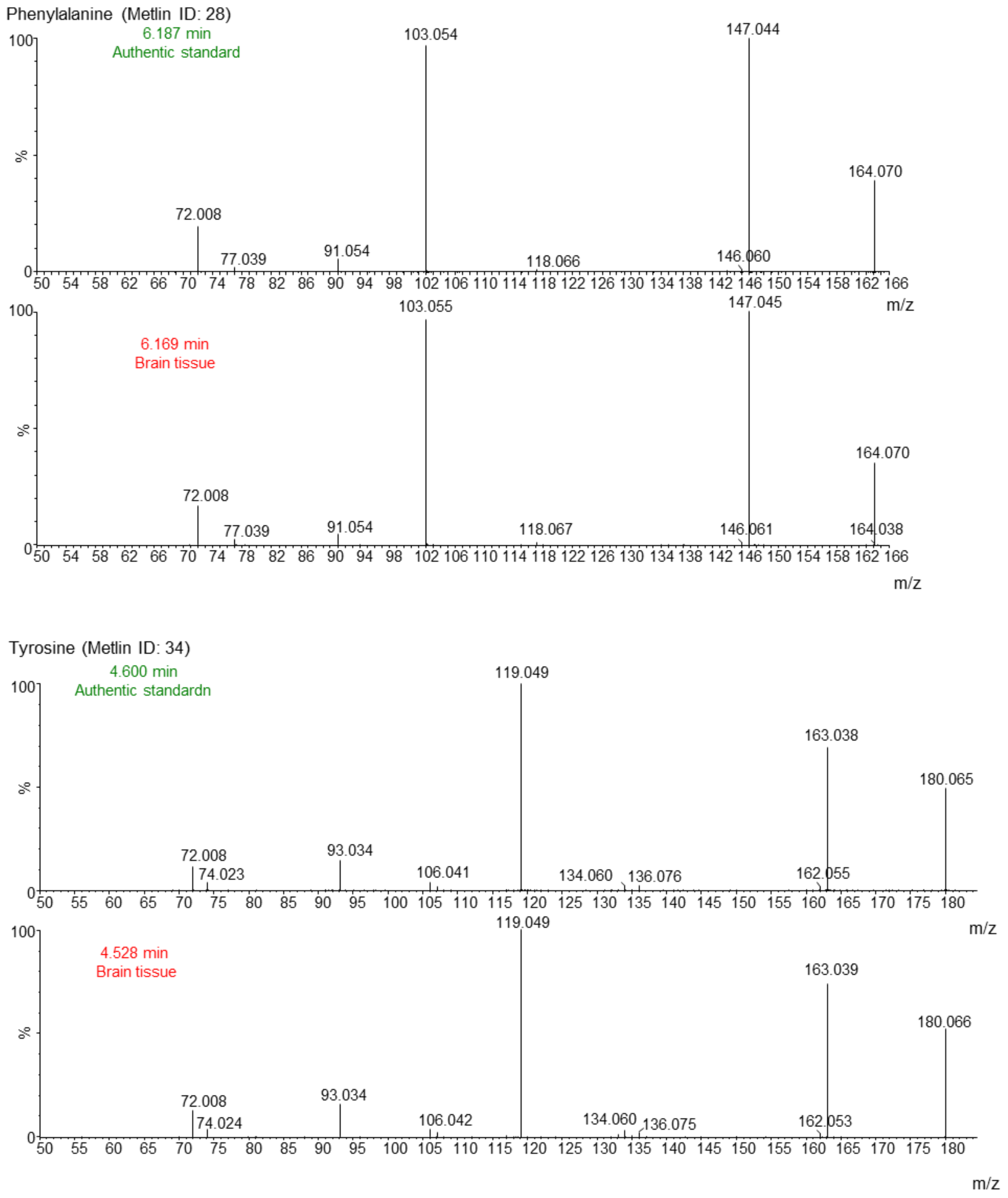

Supplementary Figure 15. Tandem MS/MS spectra for the structural validation of phenylalanine and tyrosine in negative ionization mode. Pooled brain tissue was used for the UPLC-MS/MS analysis and spectra acquired at 20 eV. The acquired MS/MS spectra were compared to MS/MS spectra collected in authentic standards. Metlin ID numbers are provided.

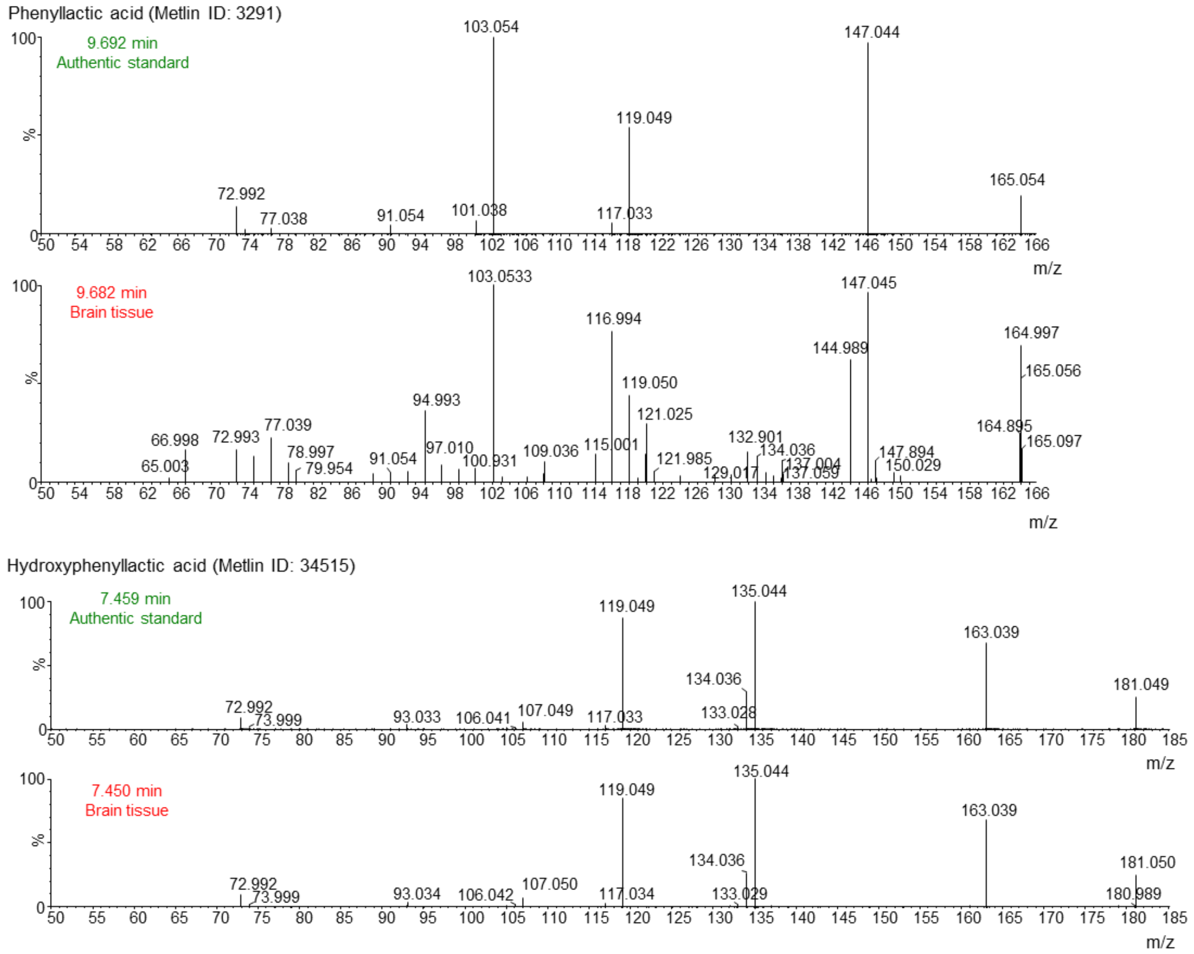

Supplementary Figure 16. Tandem MS/MS spectra for the structural validation of phenyllactic acid and hydroxyphenyllactic acid in negative ionization mode. Pooled brain tissue was used for the UPLC-MS/MS analysis and spectra acquired at 20 eV. The acquired MS/MS spectra were compared to MS/MS spectra collected in authentic standards. Metlin ID numbers are provided.

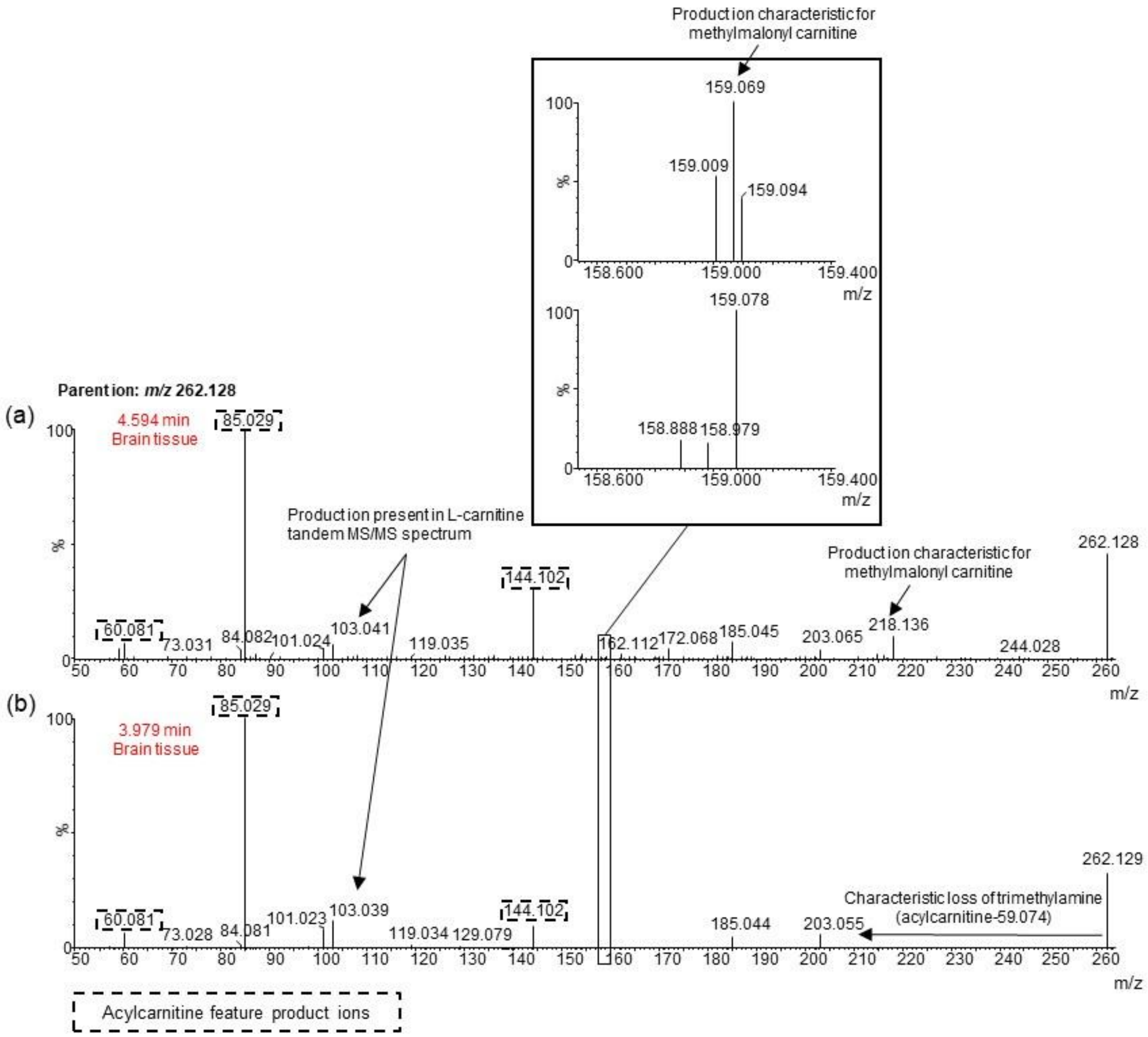

Supplementary Figure 17. Tandem MS/MS spectra for the structural validation of the *m/z* 262.128 acylcarnitines in positive ionization mode. Pooled brain tissue was used for the UPLC-MS/MS analysis and spectra acquired at 20 eV. The structure assignment was based on previous experimental data (1, 2).

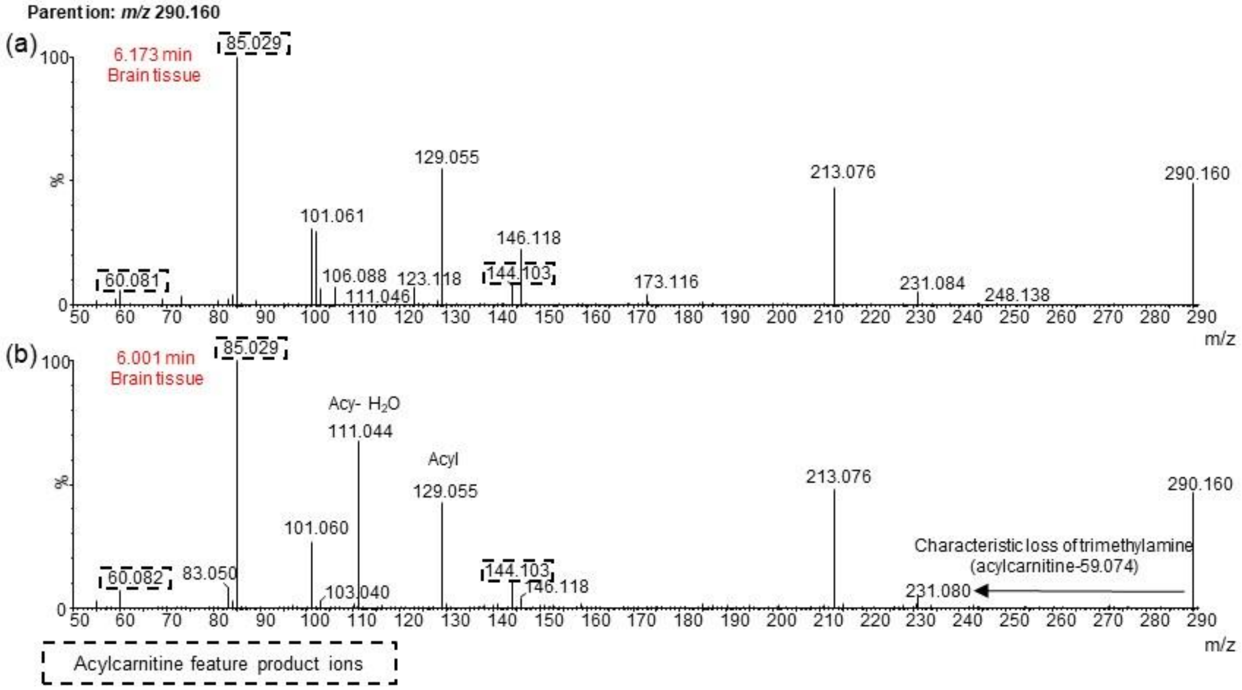

Supplementary Figure 18. Tandem MS/MS spectra for the structural validation of the *m/z* 290.160 acylcarnitines in positive ionization mode. Pooled brain tissue was used for the UPLC-MS/MS analysis and spectra acquired at 20 eV. The structure assignment was based on previous experimental data (1, 2).

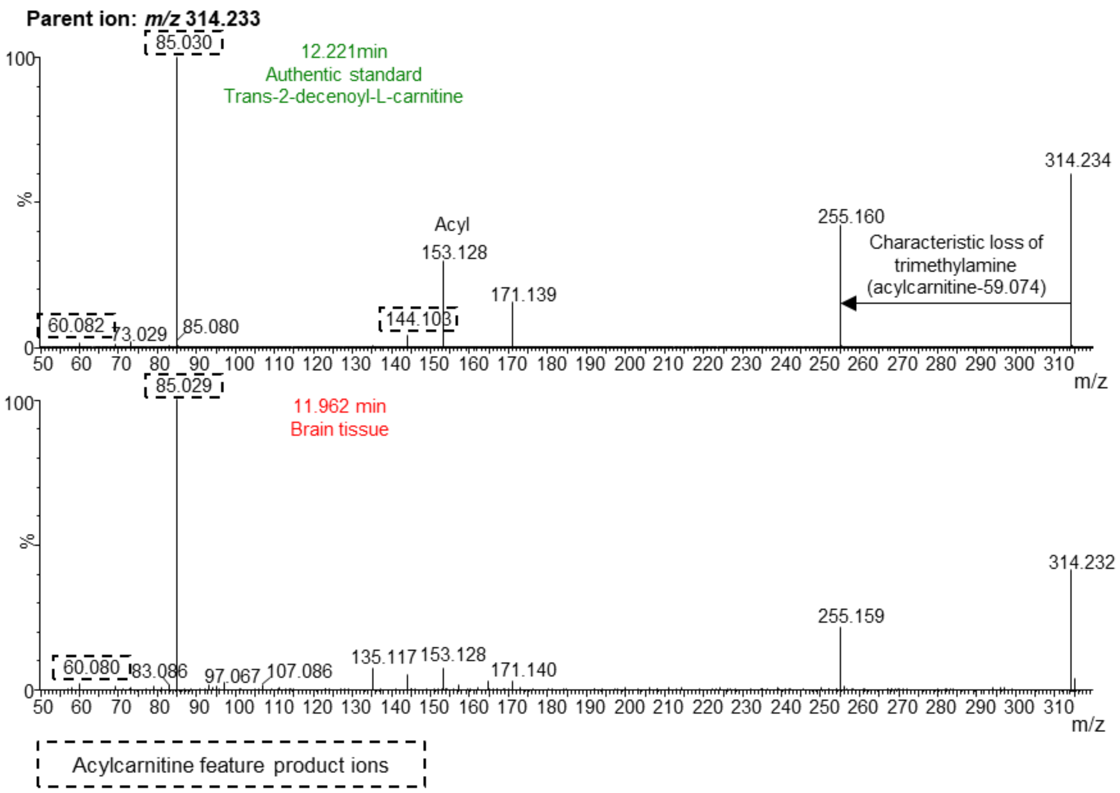

Supplementary Figure 19. Tandem MS/MS spectra for the structural validation of the *m/z* 314.233 acylcarnitine in positive ionization mode. Pooled brain tissue was used for the UPLC-MS/MS analysis and spectra acquired at 20 eV. The structure assignment was based on previous experimental data (1, 2).

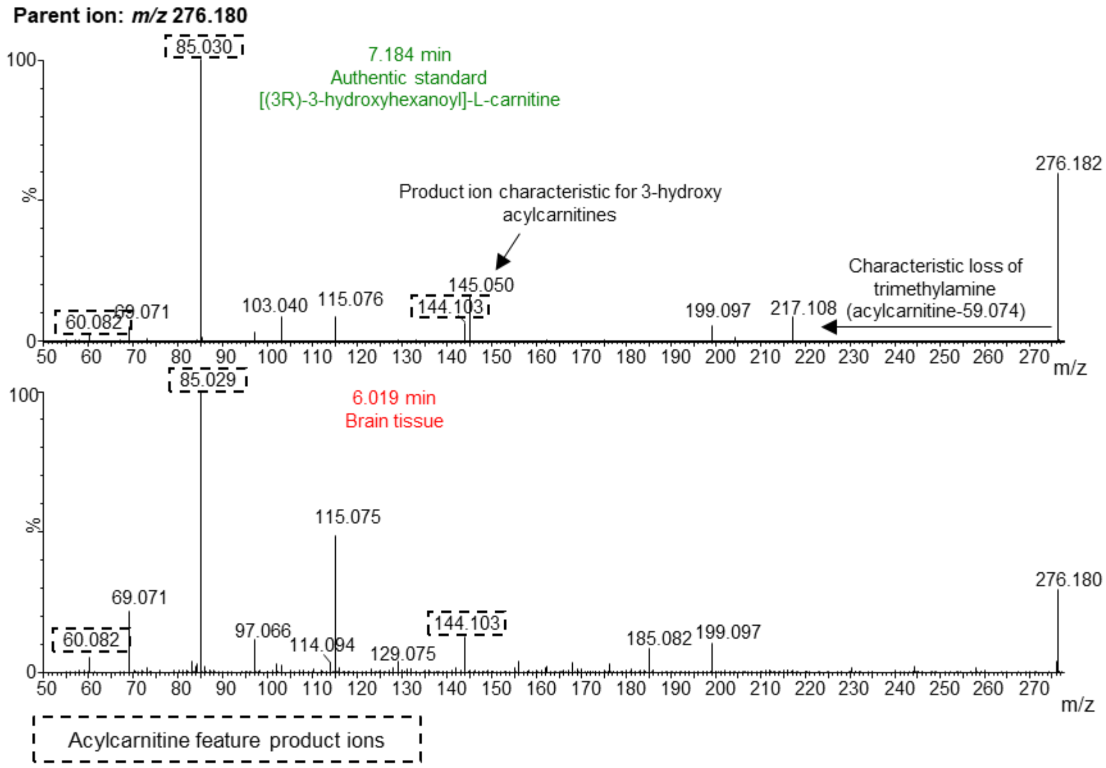

Supplementary Figure 20. Tandem MS/MS spectra for the structural validation of the *m/z* 276.180 acylcarnitine in positive ionization mode. Pooled brain tissue was used for the UPLC-MS/MS analysis and spectra acquired at 20 eV. The structure assignment was based on previous experimental data (1, 2).

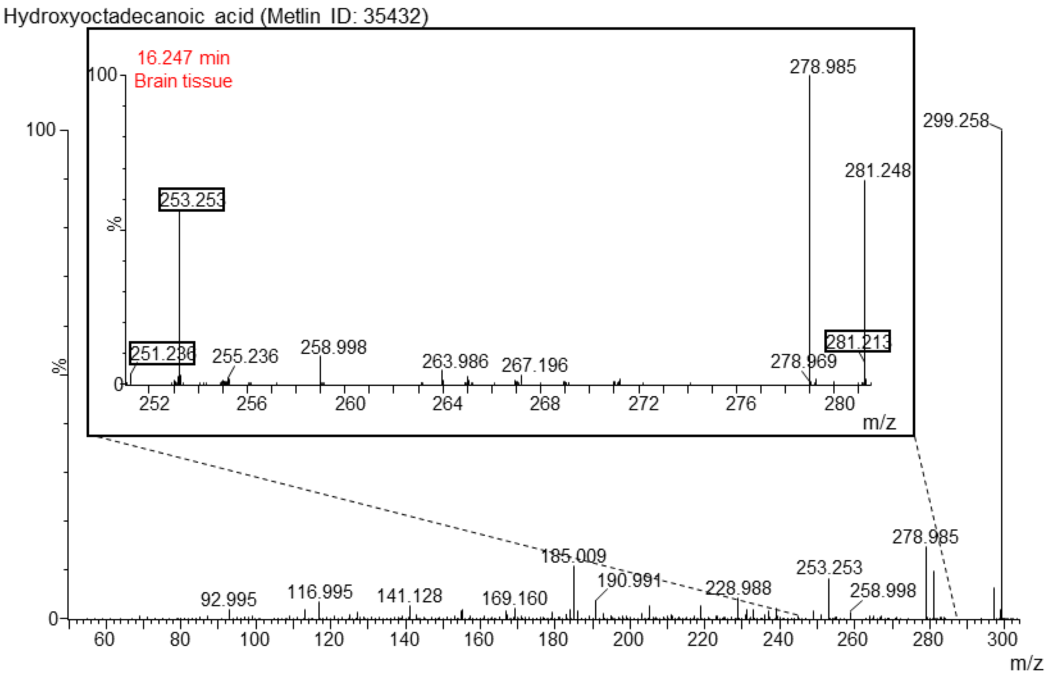

Supplementary Figure 21. Tandem MS/MS spectra for the structural validation of hydroxyoctadecanoic acid in negative ionization mode. Pooled brain tissue was used for the UPLC-MS/MS analysis and spectra acquired at 30 eV. The acquired MS/MS spectra were compared to experimental MS/MS spectra available in the Metlin database and common product ions are annotated with a black rectangle. Metlin ID numbers are provided.

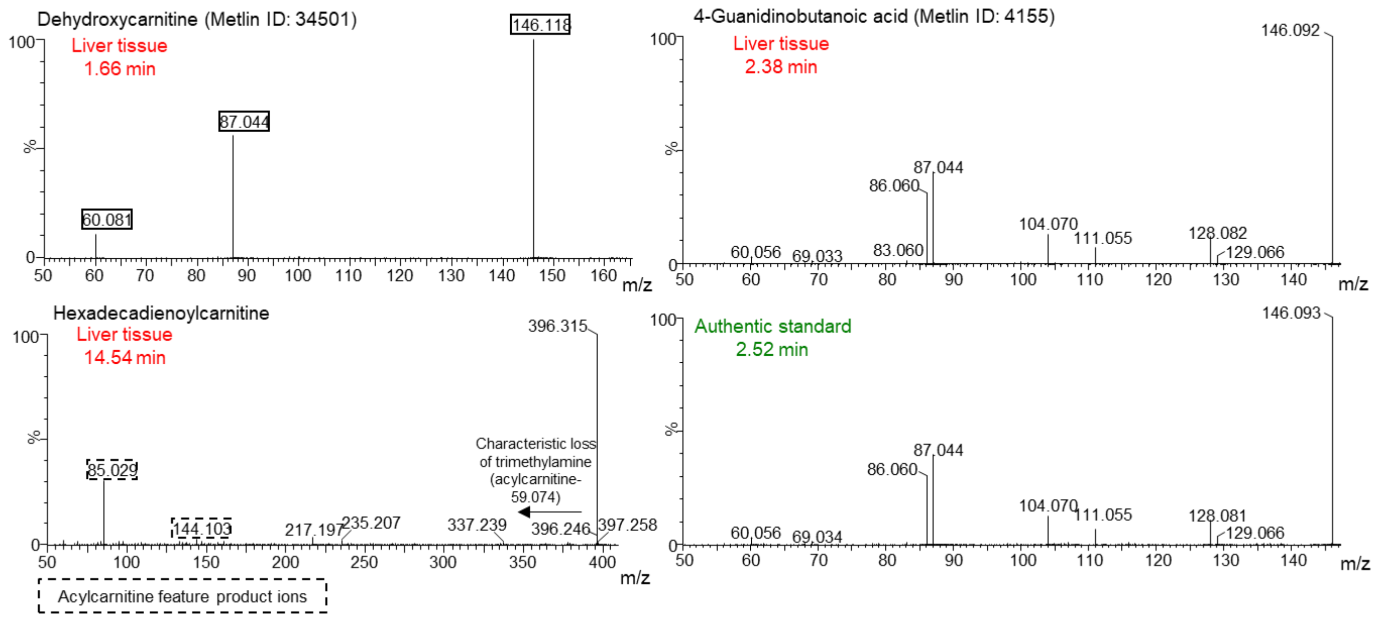

Supplementary Figure 22. Tandem MS/MS spectra for the structural validation of representative significantly altered liver metabolites in positive ionization mode. Pooled liver tissue was used for the UPLC-MS/MS analysis and spectra acquired at 10 eV. The acquired MS/MS spectra were compared to experimental MS/MS spectra available in the Metlin database and common product ions are annotated with a black rectangle. In the case of 4-Guanidinobutanoic acid, the acquired MS/MS spectra were compared to MS/MS spectra collected in authentic standards.Metlin ID numbers are provided.

Supplementary Tables

Supplementary Table 1. Evaluation of the isotope labeled internal standard in the metabolomics analysis. Values for the internal standard (C-13 isotopically labeled phenylalanine) in the quality control (QC) samples in positive (POS) and negative (NEG) ionization mode.

	**POS**	**NEG**
**Average peak area (a.u.)**	4590290	3413293
**Standard deviation**	1272127	295215
**%CV**	27.71	8.65
**RT (±SEM) (min)**	6.156 (± 0.005)	6.181(± 0.005)

Supplementary Table 2. Structure validation of the brain metabolites presented in Figure 1 and Supplementary Figure 3.

	m/z experimental	*m/z* theoretical	ppm	[a]Ionization mode	Rt brain tissue (min)	Rt authentic standard (min)	MS/MS	Confidence level*
Choline	104.107	104.1075	4.80	POS	1.41	1.41	20 eV	2a
Betaine	118.086	118.0862	-1.69	POS	1.46	1.49		2a
Citicoline	489.113	489.1146	-3.27	POS	1.70		20 eV	2a
L-carnitine	162.113	162.1124	3.70	POS	1.45	1.45	20 eV	2a
Serotonin	177.102	177.1022	-1.13	POS	5.45	5.64	20 eV	1
Creatine	132.078	132.0768	9.09	POS	1.56	1.57	20 eV	2a
Melanin	319.069	319.0713	-7.21	POS	14.30		10 eV	2a
Aspartate	132.028	132.0302	-16.66	NEG	1.36	1.34	20 eV	1
Glutamate	146.045	146.0458	-5.48	NEG	1.42	1.40	20 eV	1
Glutamine	145.060	145.0618	-12.41	NEG	1.36	1.34	20 eV	2a
Gluconic acid	195.049	195.0510	-10.25	NEG	1.44	1.44	20 eV	2a
Orotate	155.007	155.0098	-18.06	NEG	2.14	2.31	20 eV	2a
cAMP	328.044	328.0452	-3.66	NEG	5.62	5.72	20 eV	2a
Tryptophan	203.081	203.0826	-7.88	NEG	7.26	7.39	20 eV	1
N-acetyl-tryptophan	245.092	245.0931	-4.49	NEG	9.74	10.02	20 eV	1
Adenosine	266.088	266.0895	-5.64	NEG	5.40	5.42	20 eV	1
AMP	346.058	346.0558	6.36	NEG	3.12	3.18	20 eV	2a
ADP	426.020	426.0221	-4.93	NEG	4.68	4.73	20 eV	2a
Uric acid	167.019	167.0210	-11.97	NEG	3.36	3.40	20 eV	2a
N-acetyl-methionine	190.052	190.0543	12.10	NEG	7.54	7.80	20 eV	2a

Ascorbic acid	175.023	175.0248	-10.28	NEG	2.09	2.15	MSE	2a
Pantothenic acid	218.102	218.1033	-5.96	NEG	6.71	6.67	10 eV	2a

a: POS, positive ionization mode; NEG, negative ionization mode

*Confidence level: *Level 1*- the proposed structure has been confirmed via appropriate measurement of an authentic reference standard with MS, MS/MS and retention time matching; *Level 2a*- matching literature or library tandem MS/MS spectrum data where the spectrum-structure match is unambiguous (3, 4).

Supplementary Table 3. Statistical data of the sleep-wake effects on the illustrated brain metabolites in individual brain regions. Two tailed unpaired *t*-test.

	***t*- score**	***P***	**$Log_{10}FC$**
CBL_Acetyltryptophan	1.1856	0.2632	0.057
CBL_Adipoyl/methylglutarylcarnitine	1.868	0.091303	0.057
CBL_ADP	-0.029155	0.97731	-0.004
CBL_alpha-GPC	0.6724	0.51656	0.021
CBL_AMP	1.7501	0.11067	0.036
CBL_Aspartate	-1.5226	0.15883	-0.072
CBL_beta citryl glutamate	1.8396	0.09566	0.105
CBL_Betaine	1.1066	0.29437	0.052
CBL_cAMP	2.5015	0.031368	0.098
CBL_Choline	1.009	0.33677	0.036
CBL_Citicoline	-0.80921	0.43722	-0.022
CBL_Creatine	-0.18554	0.85651	-0.007
CBL_Decenoylcarnitine	4.1394	0.0020136	0.158
CBL_Ergothioneine	-3.6535	0.0044368	-0.121
CBL_Gluconic acid	-2.3004	0.044221	-0.024
CBL_Glutamate	-0.23819	0.81655	-0.004
CBL_Glutamine	1.2635	0.23506	0.025
CBL_Glutathione	-2.2868	0.045262	-0.362
CBL_Homovanillic	0.089854	0.93018	0.002
CBL_Hydroxydecanoylcarnitine	3.4775	0.0059461	0.141
CBL_Hydroxydodecanoylcarnitine	3.6465	0.0044884	0.151
CBL_Hydroxyhexanoylcarnitine	1.5012	0.16421	0.127
CBL_Hydroxyoctadecanoic acid	5.3826	0.00030894	0.218
CBL_Hydroxyphenyllactic	2.0313	0.069657	0.106
CBL_Hydroxytetradecadiencarnitine	3.3153	0.0078096	0.376
CBL_Isomer A	3.2123	0.0092951	0.080
CBL_L-carnitine	1.2094	0.25433	0.024
CBL_Melanin	3.347	0.0074033	0.152
CBL_NAAG	0.67011	0.51796	0.017
CBL_N-acetylaspartate	-0.0040951	0.99681	0.000
CBL_N-Acetyl-L-methionine	0.69493	0.50293	0.066
CBL_Orotic acid	-1.3933	0.19371	-0.071
CBL_Oxidized glutathione	2.5298	0.029881	0.049
CBL_Phenylalanine	0.60428	0.55912	0.019
CBL_Phenyllactic acid	1.4947	0.16587	0.091
CBL_SAM	-2.3102	0.043491	-0.066
CBL_Serotonin	0.16717	0.87057	0.007
CBL_S-lactoyl glutathione	3.0801	0.011638	0.269
CBL_Succinylcarnitine	-3.2086	0.0093536	-0.521
CBL_Tetradecadiencarnitine	6.5568	0.000064176	0.336
CBL_Tryptophan	1.4292	0.18343	0.059

CBL_Tyrosine	-1.355	0.20523	-0.072
CBL_Uric acid	-0.93542	0.37161	-0.051
CTX_Acetyltryptophan	1.5156	0.16057	0.088
CTX_Adipoyl/methylglutarylcarnitine	2.857	0.017044	0.113
CTX_ADP	-0.41271	0.68853	-0.039
CTX_alpha-GPC	-1.004	0.33904	-0.072
CTX_AMP	1.9159	0.084384	0.096
CTX_Aspartate	-2.3646	0.03964	-0.143
CTX_beta cityl glutamate	-2.8381	0.017604	-0.138
CTX_Betaine	-1.2469	0.24086	-0.069
CTX_cAMP	2.8721	0.016608	0.413
CTX_Choline	-0.92517	0.37666	-0.071
CTX_Citicoline	-3.6455	0.0044955	-0.098
CTX_Creatine	0.038935	0.96971	0.001
CTX_Decenoylcarnitine	4.1227	0.0020682	0.307
CTX_Ergothioneine	-1.3822	0.197	-0.062
CTX_Gluconic acid	-0.37499	0.7155	-0.009
CTX_Glutamate	1.3794	0.19783	0.022
CTX_Glutamine	1.8625	0.092137	0.054
CTX_Glutathione	-0.52707	0.60964	-0.145
CTX_Homovanillic	3.4319	0.006418	0.161
CTX_Hydroxydecanoylcarnitine	3.6597	0.0043911	0.225
CTX_Hydroxydodecanoylcarnitine	3.8429	0.0032499	0.273
CTX_Hydroxyhexanoylcarnitine	0.09276	0.92793	0.005
CTX_Hydroxyoctadecanoic acid	4.6996	0.00084213	0.451
CTX_Hydroxyphenyllactic	3.9855	0.0025779	0.141
CTX_Hydroxytetradecadiencarnitine	4.2088	0.0018032	0.313
CTX_Isomer A	5.0562	0.00049471	0.747
CTX_L-carnitine	-0.22685	0.82511	-0.007
CTX_Melanin	1.9516	0.079535	0.162
CTX_NAAG	0.69005	0.50586	0.038
CTX_N-acetylaspartate	2.9224	0.015238	0.113
CTX_N-Acetyl-L-methionine	1.6892	0.12206	0.175
CTX_Orotic acid	1.423	0.18519	0.077
CTX_Oxidized glutathione	2.3949	0.03764	0.052
CTX_Phenylalanine	0.42494	0.67988	0.010
CTX_Phenyllactic acid	2.8636	0.016852	0.132
CTX_SAM	-3.7786	0.0036104	-0.232
CTX_Serotonin	-1.2195	0.25064	-0.349
CTX_S-lactoyl glutathione	2.0881	0.063327	0.656
CTX_Succinylcarnitine	-1.529	0.15726	-0.735
CTX_Tetradecadiencarnitine	4.7914	0.00073305	0.333
CTX_Tryptophan	1.7928	0.10326	0.057
CTX_Tyrosine	0.12121	0.90593	0.010

CTX_Uric acid	-0.19457	0.84963	-0.022
HC_Acetyltryptophan	-0.71185	0.49283	-0.047
HC_Adipoyl/methylglutarylcarnitine	2.0714	0.065133	0.138
HC_ADP	-0.66049	0.52386	-0.062
HC_alpha-GPC	1.2822	0.2287	0.060
HC_AMP	0.62798	0.54409	0.023
HC_Aspartate	-1.4164	0.18705	-0.052
HC_beta citryl glutamate	0.81271	0.43531	0.151
HC_Betaine	1.6815	0.12359	0.238
HC_cAMP	-0.31344	0.76039	-0.034
HC_Choline	1.1316	0.28423	0.063
HC_Citicoline	-0.4253	0.67962	-0.010
HC_Creatine	1.0913	0.30075	0.091
HC_Decenoylcarnitine	2.2411	0.048912	0.233
HC_Ergothioneine	-0.30523	0.76645	-0.024
HC_Gluconic acid	0.96648	0.3566	0.027
HC_Glutamate	0.44154	0.66822	0.011
HC_Glutamine	0.72336	0.48603	0.017
HC_Glutathione	3.4188	0.0065608	0.401
HC_Homovanillic	-0.039322	0.96941	-0.001
HC_Hydroxydecanoylcarnitine	-1.2636	0.23505	-0.088
HC_Hydroxydodecanoylcarnitine	2.6536	0.024164	0.244
HC_Hydroxyhexanoylcarnitine	3.8665	0.003127	0.341
HC_Hydroxyoctadecanoic acid	1.798	0.10238	0.240
HC_Hydroxyphenyllactic	1.6229	0.13568	0.095
HC_Hydroxytetradecadiencarnitine	1.5528	0.15153	0.266
HC_Isomer A	1.9395	0.081142	0.082
HC_L-carnitine	0.75298	0.46882	0.048
HC_Melanin	0.80499	0.43954	0.047
HC_NAAG	0.83596	0.4227	0.030
HC_N-acetylaspartate	0.59904	0.56247	0.060
HC_N-Acetyl-L-methionine	1.6511	0.12974	0.142
HC_Orotic acid	-1.0125	0.33519	-0.136
HC_Oxidized glutathione	1.1233	0.28754	0.028
HC_Phenylalanine	0.95148	0.36379	0.025
HC_Phenyllactic acid	1.1731	0.26796	0.074
HC_SAM	0.36491	0.72278	0.013
HC_Serotonin	0.77093	0.45858	0.052
HC_S-lactoyl glutathione	3.366	0.0071693	0.606
HC_Succinylcarnitine	0.26367	0.79739	0.053
HC_Tetradecadiencarnitine	1.1826	0.26433	0.155
HC_Tryptophan	1.897	0.087049	0.082
HC_Tyrosine	0.26262	0.79817	0.013
HC_Uric acid	0.22032	0.83005	0.014

MDB_Acetyltryptophan	-0.73033	0.48195	0.009
MDB_Adipoyl/methylglutarylcarnitine	-0.22495	0.82655	0.050
MDB_ADP	1.1164	0.29035	0.224
MDB_alpha-GPC	-0.52527	0.61084	0.028
MDB_AMP	0.40629	0.69309	0.131
MDB_Aspartate	-1.4729	0.17155	-0.045
MDB_beta citryl glutamate	-0.72865	0.48293	-0.044
MDB_Betaine	-0.7901	0.4478	-0.010
MDB_cAMP	-0.99295	0.34415	-0.057
MDB_Choline	-0.25877	0.80106	0.047
MDB_Citicoline	-0.62603	0.54532	0.018
MDB_Creatine	-1.3571	0.20459	-0.043
MDB_Decenoylcarnitine	1.3626	0.2029	0.297
MDB_Ergothioneine	-1.9732	0.076738	-0.111
MDB_Gluconic acid	-1.0804	0.30534	-0.006
MDB_Glutamate	-0.39048	0.70437	0.037
MDB_Glutamine	-0.65972	0.52434	0.017
MDB_Glutathione	-0.1118	0.91319	0.060
MDB_Homovanillic	-0.57534	0.57778	0.022
MDB_Hydroxydecanoylcarnitine	0.02921	0.97727	0.073
MDB_Hydroxydodecanoylcarnitine	0.52903	0.60833	0.154
MDB_Hydroxyhexanoylcarnitine	1.4846	0.16846	0.283
MDB_Hydroxyoctadecanoic acid	0.45552	0.65846	0.131
MDB_Hydroxyphenyllactic	0.47011	0.64836	0.112
MDB_Hydroxytetradecadiencarnitine	-0.47643	0.64401	-0.009
MDB_Isomer A	0.72905	0.4827	0.155
MDB_L-carnitine	-1.4793	0.16987	-0.054
MDB_Melanin	-3.1152	0.010963	-0.376
MDB_NAAG	-1.0363	0.32447	-0.013
MDB_N-acetylaspartate	-0.23615	0.81809	0.004
MDB_N-Acetyl-L-methionine	-2.7163	0.021698	-0.274
MDB_Orotic acid	0.189	0.85387	0.100
MDB_Oxidized glutathione	-1.3728	0.19981	-0.032
MDB_Phenylalanine	-0.95476	0.36221	-0.002
MDB_Phenyllactic acid	-0.65239	0.52886	0.009
MDB_SAM	-1.3617	0.20317	-0.027
MDB_Serotonin	0.13043	0.89881	0.097
MDB_S-lactoyl glutathione	-2.1789	0.05435	-0.490
MDB_Succinylcarnitine	-1.7243	0.11537	-0.328
MDB_Tetradecadiencarnitine	-0.13082	0.89851	0.053
MDB_Tryptophan	-0.11935	0.90736	0.057
MDB_Tyrosine	0.10176	0.92096	0.093
MDB_Uric acid	1.4372	0.1812	0.294

logFC: Log_{10}(fold change); fold change = (Average sleep-state)/ (Average wake-state)

Supplementary Table 4. Structure validation of the sulfur containing brain metabolites presented in Figure 2. Tandem MS/MS spectra were acquired in positive ionization mode.

	***m/z* experimental**	***m/z* theoretical**	**ppm**	**Rt brain tissue (min)**	**Rt authentic standard (min)**	**MS/MS**	**Confidence level***
Glutathione	308.092	308.0911	2.92	2.8	2.8	20 eV	1
Oxidized glutathione	613.163	613.1592	6.20	4.92	5.07	20 eV	1
SAM	399.144	399.145	-2.51	1.49	1.51	20 eV	1
S-lactoylglutathione	380.112	380.1122	-0.53	5.29		20 eV	2a

*Confidence level: Level 1- the proposed structure has been confirmed via appropriate measurement of an authentic reference standard with MS, MS/MS and retention time matching; Level 2a- matching literature or library tandem MS/MS spectrum data where the spectrum-structure match is unambiguous *(3, 4)*.

Supplementary Table 5. Structure validation of the brain metabolites presented in Figure 3. Tandem MS/MS spectra were acquired in negative ionization mode.

	***m/z* experimental**	***m/z* theoretical**	**ppm**	**Rt brain tissue (min)**	**Rt authentic standard (min)**	**MS/MS**	**Confidence level***
NAAG	303.082	303.0834	-4.62	4.78		20eV	2a
N-acetylaspartate	174.040	174.0408	-4.60	2.75	2.80		2a

Confidence level: Level 1*- the proposed structure has been confirmed via appropriate measurement of an authentic reference standard with MS, MS/MS and retention time matching; *Level 2a*- matching literature or library tandem MS/MS spectrum data where the spectrum-structure match is unambiguous (3, 4).

Supplementary Table 6. Structure validation of the phenylalanine and tyrosine brain metabolites presented in Figure 4. Tandem MS/MS spectra were acquired in negative ionization mode.

	***m/z* experimental**	***m/z* theoretical**	**ppm**	**Rt brain tissue (min)**	**Rt authentic standard (min)**	**MS/MS**	**Confidence level***
Tyrosine	180.065	180.0665	-8.33	4.58	4.72	20 eV	1
Phenylalanine	164.070	164.0716	-9.75	6.16	6.16	20 eV	1
Phenyllactic acid	165.054	165.0556	-9.69	9.68	9.74	20 eV	1
Hydroxyphenyllactic acid	181.049	181.0506	-8.84	7.45	7.75	20 eV	1
Homovanillic acid	181.048	181.0506	-14.36	8.62	8.65	20 eV	1

*Confidence level: *Level 1*- the proposed structure has been confirmed via appropriate measurement of an authentic reference standard with MS, MS/MS and retention time matching; *Level 2a*- matching literature or library tandem MS/MS spectrum data where the spectrum-structure match is unambiguous (3, 4).

Supplementary Table 7. Structure validation of the acylcarnitines presented in Figure 5.

	***m/z* experimental**	***m/z* theoretical**	**ppm**	**Ionization mode**	**Rt brain tissue (min)**	**Rt authentic standard (min)**	**MS/MS**	**Confidence level***
Succinylcarnitine	262.128	262.1285	-1.91	POS	4.08		20eV	2a
2-Hydroxyhexanoylcarnitine	276.180	276.1805	-1.81	POS	6.00		20eV	2a
Adipoyl/methylglutarylcarnitine	290.159	290.1598	-2.76	POS	5.98		20eV	2a
Decenoylcarnitine	314.233	314.2326	1.27	POS	12.01	12.27	20eV	1
3-Hydroxydecanoylcarnitine	332.243	332.2431	-0.30	POS	11.64		20eV	2a
Hydroxydodecanoylcarnitine	360.275	360.2744	1.67	POS	12.08		20eV	2a
Tetradecadiencarnitine	368.283	368.2795	9.50	POS	13.55		20eV	2a
3-Hydroxytetradecadiencarnitine	384.276	384.2744	4.16	POS	12.91		20eV	2a
Hydroxyoctadecanoic acid	299.258	299.2592	-4.01	NEG	16.07		30eV	2a

a: POS, positive ionization mode; NEG, negative ionization mode
*Confidence level: *Level 1*- the proposed structure has been confirmed via appropriate measurement of an authentic reference standard with MS, MS/MS and retention time matching; *Level 2a*- matching literature or library tandem MS/MS spectrum data where the spectrum-structure match is unambiguous (3, 4).

Supplementary Table 8. Two-way ANOVA results of the illustrated brain metabolites.

	F-statistic sleep-wake effect	*P* value (raw)	FDR	*F*-statistic brain region effect	*P* value (raw)	FDR	*F*-statistic interaction	*P* value (raw)	FDR
Cityl glutamate isomer A	27.79	0.00001	0.00114	47.51	4.29E-13	5.85E-12	24.47	4.51E-09	0.00002
Homovanillic acid	10.34	0.00262	0.11950	50.92	1.48E-13	2.24E-12	7.84	0.00033	0.04194
Hydroxyphenyllactic acid	26.48	0.00001	0.00159	53.66	6.58E-14	1.09E-12	2.23	0.09983	0.39346
Aspartate	8.98	0.00473	0.16384	81.32	7.80E-17	2.67E-15	0.07	0.97602	0.99522
Glutamine	4.14	0.04873	0.43054	10.05	0.00005	0.00011	0.44	0.72725	0.89176
Choline	1.00	0.32368	0.77344	13.92	2.56E-06	0.00001	1.30	0.28818	0.61582
Glutamate	2.76	0.10486	0.55945	9.61	0.00007	0.00015	0.68	0.56984	0.80624
Gluconic acid	0.20	0.65651	0.91483	78.49	1.41E-16	4.48E-15	1.39	0.26155	0.59097
L-carnitine	0.10	0.75436	0.94486	89.12	1.67E-17	6.85E-16	1.06	0.37600	0.68500
Creatine	0.19	0.66809	0.91906	1.97	0.13454	0.15388	1.00	0.40516	0.70506
Orotate	0.15	0.70071	0.92727	20.61	3.65E-08	1.57E-07	1.69	0.18594	0.51119
Adenosine	1.659	0.2054	0.330	18.27	0.0001	8.43E-4	3.560	0.0227	0.1316
AMP	8.75	0.00525	0.17011	10.87	0.00003	0.00006	0.87	0.46550	0.74438
Uric acid	0.69	0.40966	0.82199	4.98	0.00506	0.00763	2.00	0.12978	0.43778
ADP	0.03	0.87141	0.97041	4.75	0.00645	0.00956	0.70	0.55549	0.79750
Serotonin	0.16	0.69111	0.92404	6.30	0.00136	0.00228	1.26	0.30215	0.62734
cAMP	8.23	0.00662	0.19043	10.08	0.00005	0.00010	5.44	0.00318	0.10660
Tryptophan	10.85	0.00211	0.10500	55.16	4.27E-14	7.35E-13	0.12	0.94984	0.98794
N-acetyltryptophan	3.18	0.08255	0.51905	55.73	3.63E-14	6.33E-13	1.22	0.31700	0.64036
Melanin	0.96	0.33256	0.77946	24.55	4.33E-09	2.28E-08	9.49	0.00008	0.01991
Betaine	0.83	0.36751	0.80132	46.96	5.12E-13	6.87E-12	1.54	0.21908	0.54814
Citicoline	3.61	0.06483	0.47575	5.69	0.00249	0.00396	1.01	0.39981	0.70172
N-acetylmethionine	1.92	0.17358	0.64775	17.02	3.18E-07	1.10E-06	2.95	0.04471	0.28522
Ascorbic acid	1.42	0.24063	0.71466	3.37	0.02806	0.03683	1.32	0.28015	0.60849
Pantothenic acid	0.19	0.66546	0.91810	87.32	2.35E-17	9.29E-16	0.05	0.98627	0.99720
Ergothioneine	13.52	0.00071	0.05348	75.63	2.62E-16	7.76E-15	2.42	0.08068	0.35945

Glutathione	0.27	0.60413	0.89859	3.57	0.02241	0.03002	5.18	0.00413	0.11693
Oxidized glutathione	5.60	0.02306	0.33037	4.32	0.01012	0.01447	2.32	0.09025	0.37710
SAM	14.24	0.00054	0.04342	82.26	6.43E-17	2.25E-15	3.09	0.03792	0.26705
S-lactoyl glutathione	12.54	0.00105	0.06815	65.55	2.72E-15	6.21E-14	7.23	0.00057	0.05324
β-citryl glutamate	0.01	0.90490	0.97830	21.92	1.75E-08	8.10E-08	2.17	0.10668	0.40410
NAAG	0.24	0.62948	0.90645	96.38	4.39E-18	2.10E-16	0.28	0.84268	0.94581
N-acetylasparate	1.03	0.31695	0.76967	5.19	0.00409	0.00627	0.25	0.85890	0.95352
Phenylalanine	0.82	0.37174	0.80390	142.82	4.50E-21	4.81E-19	0.10	0.95986	0.99077
Tyrosine	0.03	0.87007	0.97038	1.65	0.19450	0.21548	0.65	0.59002	0.81701
Phenyllactic acid	9.47	0.00381	0.14415	30.89	2.14E-10	1.53E-09	2.02	0.12665	0.43255
Succinylcarnitine	11.28	0.00176	0.09441	5.55	0.00287	0.00452	2.60	0.06585	0.33125
Adipoyl-methylglutarylcarnitine	10.18	0.00280	0.12492	16.85	3.55E-07	1.21E-06	0.68	0.56708	0.80436
Decenoylcarnitine	35.42	0.00000	0.00018	3.06	0.03939	0.05023	0.31	0.81710	0.93507
Tetradecadiencarnitine	17.88	0.00014	0.01725	1.25	0.30436	0.32539	2.01	0.12782	0.43455
2-hydroxyhexanoylcarnitine	16.00	0.00027	0.02785	7.73	0.00036	0.00067	2.62	0.06421	0.32785
3-hydroxydecanoylcarnitine	10.56	0.00238	0.11356	6.90	0.00078	0.00136	5.45	0.00317	0.10660
Hydroxydodecanoylcarnitine	23.52	0.00002	0.00350	3.89	0.01595	0.02199	0.28	0.83991	0.94494
3-hydroxytetradecadiencarnitine	9.08	0.00452	0.15944	3.21	0.03334	0.04313	1.55	0.21653	0.54622
Hydroxyoctadecanoic acid	21.00	0.00005	0.00693	5.38	0.00338	0.00526	1.25	0.30497	0.63010

FDR: false discovery rate (adjusted *P* value)

Supplementary Table 9. Statistical data of the illustrated metabolites in plasma.

	m/z	Rt (min)	Mode	*P* value	t-score	CI (95%)
Choline	104.106	1.45	POS	0.7149	0.3759	-0.2757 to 0.1961
Betaine	118.086	1.49	POS	0.4816	0.7309	-0.6984 to 0.3534
L-carnitine	162.113	1.51	POS	0.6147	0.5196	-3.075 to 1.912
Serotonin	177.101	5.67	POS	0.3473	0.9863	-0.07081 to 0.1833
Creatine	132.077	1.63	POS	0.4816	0.7310	-4.088 to 2.068
Aspartate	132.029	1.28	NEG	0.9538	0.05938	-0.008721 to 0.009199
Glutamate	146.045	1.42	NEG	0.4702	0.7506	-0.03144 to 0.01560
Glutamine	145.060	1.39	NEG	0.2048	1.356	-0.08427 to 0.02049
Gluconic acid	195.050	1.42	NEG	0.6554	0.4599	-0.2851 to 0.1876
Orotate	155.008	2.26	NEG	0.6956	0.4028	-0.002736 to 0.001898
cAMP	328.044	5.76	NEG	0.1651	1.498	-0.03798 to 0.007445
Tryptophan	203.082	7.34	NEG	0.7171	0.3728	-3.819 to 2.724
N-acetyltryptophan	245.092	9.90	NEG	0.8697	0.1683	-0.4399 to 0.5118
N-acetylmethionine	190.053	7.55	NEG	0.9337	0.08528	-0.2568 to 0.2379
Succinylcarnitine	262.128	4.17	POS	0.4689	0.7528	-0.4296 to 0.2126
Decenoylcarnitine	314.233	12.25	POS	0.1499	1.560	-0.0530 to 0.3003
Tetradecadiencarnitine	368.281	13.77	POS	0.1019	1.801	-0.0288 to 0.2713
2-hydroxyhexanoylcarnitine	276.179	6.15	POS	0.6755	0.4311	-0.1551 to 0.1048
Hydroxyoctadecanoic acid	299.259	16.15	NEG	0.2383	1.254	-0.0532 to 0.1901

Table 10. Statistical data of the illustrated metabolites in liver

	m/z	Rt (min)	Mode	*P* value	t-score
4-Guanidinobutanoic acid	146.093	2.28	POS	8.43000E-07	-4.6986
Creatine	132.077	1.64	POS	0.00023	4.2631
Dehydroxycarnitine	146.118	1.67	POS	0.00033	4.2129
SAM	399.145	1.56	POS	0.00035	4.2050
N-Acetyl-L-arginine	217.130	2.29	POS	0.00040	-4.1856
Tetradecadiencarnitine	368.282	13.48	POS	0.00070	4.0968
Tetradecenoylcarnitine	370.297	13.89	POS	0.0038	3.7897
Hexadecadienoylcarnitine	396.3113	14.11	POS	0.0062	3.6111
Hexadecenoylcarnitine	398.3277	14.442	POS	0.0029	3.8071
Oxoarginine	174.087	2.05	POS	0.00629	-3.6087
Betaine	118.087	1.50	POS	0.01298	-3.3813
α-GPC	258.111	1.44	POS	0.01866	3.2504
Gluconic acid	195.050	1.43	NEG	0.02011	-3.2218
Serotonin	177.102	5.57	POS	0.02313	3.1667
Choline	104.107	1.43	POS	0.03374	3.0072
Glutamate	146.044	1.46	NEG	0.03676	2.9686
Aspartate	132.029	1.39	NEG	0.05676	2.7572
N-Acetyl-methionine	190.053	7.74	NEG	0.09514	-2.4667
ADP	426.020	4.72	NEG	0.11157	2.3670
Hydroxyphenyllactic acid	181.049	7.61	NEG	0.14236	-2.2035
Glutamine	145.060	1.39	NEG	0.17390	2.0582
Adipoylcarnitine/ Methylglutarylcarnitine	290.161	6.09	POS	0.19781	1.9588
Oxidized glutathione	613.162	5.09	POS	0.20650	1.9246
N-acetyl-aspartate	174.039	2.91	NEG	0.23188	1.8292
Uric acid	167.020	3.58	NEG	0.28937	-1.6340
Hydroxydodecanoyl carnitine	360.275	12.05	POS	0.36004	-1.4221
Hydroxy-Octadecanoic acid	299.258	16.20	POS	0.41339	-1.2765
Xanthine	151.025	4.63	NEG	0.44025	-1.2067
Pyroglutamate	128.034	3.52	NEG	0.47882	1.1101
L-Tyrosine	180.065	4.83	NEG	0.48782	-1.0880
Glutathione	308.092	2.61	POS	0.53102	0.9848
Succinylcarnitine	262.129	4.23	POS	0.61790	-0.7871
L-Phenylalanine	164.070	6.37	NEG	0.64400	0.7299
Phenyllactic acid	165.054	9.93	NEG	0.67566	-0.6615
Pantothenic acid	218.102	6.87	NEG	0.68064	-0.6508
AMP	346.055	3.36	NEG	0.69202	-0.6266
Uridine	243.061	4.96	NEG	0.72765	-0.5514
N-acetyltryptophan	245.092	9.94	NEG	0.72943	-0.5476
Citicoline	489.114	1.69	POS	0.73858	-0.5285
Hydroxyhexanoycarnitine	276.181	6.12	POS	0.76397	-0.4758
S-Lactoylglutathione	380.110	5.44	POS	0.80811	-0.3852
Cyclic AMP	328.044	5.73	NEG	0.82529	-0.3502
L-Carnitine	162.113	1.48	POS	0.84454	0.3111

L-Tryptophan	203.081	7.39	NEG	0.85752	0.2849
Ascorbic acid	175.023	2.21	NEG	0.98073	0.0384

SI References

1. Yu D, Zhou L, Xuan Q, Wang L, Zhao X, Lu X, et al. Strategy for Comprehensive Identification of Acylcarnitines Based on Liquid Chromatography-High-Resolution Mass Spectrometry. Anal Chem. 2018;90(9):5712-8.
2. Yan X, Markey SP, Marupaka R, Dong Q, Cooper BT, Mirokhin YA, et al. Mass Spectral Library of Acylcarnitines Derived from Human Urine. Anal Chem. 2020;92(9):6521-8.
3. Schymanski EL, Jeon J, Gulde R, Fenner K, Ruff M, Singer HP, et al. Identifying small molecules via high resolution mass spectrometry: communicating confidence. Environ Sci Technol. 2014;48(4):2097-8.
4. Correia MSP, Jain A, Alotaibi W, Young Tie Yang P, Rodriguez-Mateos A, Globisch D. Comparative dietary sulfated metabolome analysis reveals unknown metabolic interactions of the gut microbiome and the human host. Free Radic Biol Med. 2020;160:745-54.
